# The combination treatment of RC48 and STAT3 inhibitor acts as a promising therapeutic strategy for basal bladder cancer

**DOI:** 10.3389/fimmu.2024.1432586

**Published:** 2025-01-07

**Authors:** Jingxian Li, Kun Shan, Wei Huang, Qiang Su, Yuanjiong Qi, Zhihong Zhang, Jianqiang Zhu, E. Du

**Affiliations:** Tianjin Institute of Urology, The Second Hospital of Tianjin Medical University, Tianjin, China

**Keywords:** RC48-ADC, MIBC, artesunate, molecular subtypes, targeted therapy

## Abstract

As an antibody-drug conjugate (ADC), disitamab vedotin (RC48) is a promising treatment targeting ERBB2 for locally advanced and metastatic bladder cancer (BLCA). However, the subtype heterogeneity of muscle-invasive bladder cancer (MIBC) often leads to different therapeutic outcomes. In our study, we aim to explore sensitivity differences and mechanisms of different molecular subtypes of MIBC to RC48 treatment and develop a strategy for combination therapy against cancer. Using large-scale mRNA expression profile datasets, Western blotting, and immunohistochemistry, we first found that ERBB2 is upregulated in the luminal type but downregulated in basal bladder cancer. In addition, luminal cells showed higher sensitivity to RC48 than basal cells. Basal cells with ERBB2 overexpression demonstrated increased sensitivity to RC48 *in vitro* and *in vivo*, indicating that ERBB2 expression mediates RC48’s therapeutic efficacy against cancer. In basal or RC48-exposed luminal cells, the JAK/STAT3 pathway was highly enriched, suggesting that downregulation or pharmacological inhibition of ERBB2 leads to compensatory activation of this pathway. Silencing STAT3 increased the inhibitory efficacy of RC48. In addition, artesunate (ART, a STAT3 inhibitor) showed a synergistic effect with RC48 against basal bladder cancer both *in vitro* and *in vivo*. In summary, these findings provide a theoretical foundation for subsequent clinical trials combining RC48 and ART in MIBC based on molecular subtypes.

## Introduction

As one of the most common urological malignancies, bladder cancer (BLCA) has approximately 573,278 new cases in 2020, and this number is expected to double by 2040 according to World Health Organization predictions (1). BLCA mainly contains muscle-invasive bladder cancer (MIBC), nonmuscle-invasive bladder cancer (NMIBC), and metastatic bladder cancer (2). For advanced MIBC, the primary therapy options include surgery, chemotherapy, and immunotherapy (3). Although cisplatin-based chemotherapy has made a remarkable achievement in reducing the risk of BLCA-induced death, the overall response rate is still low, approximately less than 50% (2). For patients ineligible for cisplatin, immunotherapy is the first-line treatment recommended for those with positive expression of programmed death ligand-1 (PD-L1) (3). However, the complete and partial response rates of this treatment remain low (4). Of note, due to the limitations of current therapeutic methods, novel therapeutic strategies must still be explored.

The development of ADC made a promising treatment strategy for bladder cancer, demonstrating improved overall survival in patients (5). Enfortumab vedotin (EV) was the first ADC to report encouraging results. Patients treated with EV and pembrolizumab showed longer progression-free survival compared to those treated with chemotherapy (6). Another new ADC, sacituzumab govitecan (SG), shows a promise as a treatment strategy for BLCA by targeting trophoblast cell surface antigen 2 (Trop-2), with an ORR of 27% against metastatic bladder cancer (7). RC48 is an ADC targeting ERBB2 and has been reported as a promising therapeutic method for treating various cancers, including breast cancer (8), gastric cancer (9), non-small-cell lung cancer (10), and bladder cancer (11). A multicenter phase II study revealed that 51.2% of advanced urothelial carcinoma patients received a partial response, none of them received a complete response, and the rest represented stable disease (39.5%) and disease progression (7%) (11). These data suggested a feasible therapeutic strategy for RC48 against urothelial carcinoma. Of note, not all patients can receive the efficient clinical benefits treated with these ADCs due to tumor heterogeneity and individual differences.

Recently, MIBC consensus subtypes were established based on a prior classification system, dividing MIBC into six subtypes: basal/squamous (Ba/Sq), luminal unstable (LumU), luminal nonspecified (LumNS), luminal papillary (LumP), neuroendocrine-like (NE-like), and stroma-rich (12). This classification provides a robust foundation and framework for testing and validating potential biomarkers in future clinical trials. In addition, MIBC cells have been classified into basal and luminal types (13), offering a foundation for subsequent preclinical trials. Different molecular subtypes of BLCA show heterogeneous sensitivity for ADC. For example, the sensitivity of EV is mediated by the expression of NECTIN4, which is highly expressed in luminal BLCA (14). In contrast, TROP2 expression is higher in bladder cancer cells resistant to EV, which remain sensitive to SG (15). However, the sensitivity of different molecular subtypes of MIBC to RC48 remains unclear. Given the different therapeutic responses observed in patients treated with RC48, it is necessary to explore the potential reasons for treatment failure based on MIBC molecular subtypes.

Our study found that ERBB2, the target of RC48, is highly enriched in luminal bladder cancer. Luminal bladder cancer cells demonstrated higher sensitivity to RC48 compared to basal bladder cancer cells. Moreover, we observed significant enrichment of the JAK/STAT3 pathway in basal bladder cancer. In RC48-treated bladder cancer cells, compensatory activation of the JAK/STAT3 pathways was noted. Pharmacological inhibition of STAT3 by ART could overcome the resistance of basal cells to RC48. Overall, this study provides a foundation for developing clinical therapeutic strategies combining STAT3 inhibitors with RC48 in MIBC.

## Materials and methods

### Public available data collection and subsequent analyses

The Cancer Genome Atlas (TCGA)-MIBC mRNA expression profile was obtained from the UCSC Xena public website (http://xena.ucsc.edu/). Gene expression values were normalized and transformed into LCPM units using the edgeR package. Additionally, normalized mRNA expression data from GSE13507, GSE32894, GSE48075, GSE48276, and single-cell RNA-sequencing data from GSE135337 were retrieved from the Gene Expression Omnibus (GEO) database (https://www.ncbi.nlm.nih.gov/gds). RNA-sequencing data for cell lines representing bladder cancer were obtained from the Cancer Cell Line Encyclopedia (CCLE) database (https://sites.broadinstitute.org/ccle/). TCGA-MIBC protein expression profile was collected from The Cancer Proteome Atlas (TCPA) database (https://tcpaportal.org/tcpa/). The six consensus subtypes of TCGA-MIBC—Ba/Sq, LumU, LumNS, LumP, NE.like, and stroma-rich—were defined based on previous literature (12). Other classification methods, including Baylor types, UNC types, Lund types, TCGA types, and MDA types, were determined by the BLCAsubtyping R-package (12). Bladder cancer cells from the CCLE were categorized into two types—luminal and basal—based on previous research (13). ERBB2 expression values were extracted from these datasets to compare the expression differences among subtypes.

### Differential expression genes screening and weighted coexpression network analysis

To identify differentially expressed genes (DEGs) in MIBC compared to normal tissues, we performed differential expression analysis based using the limma R-package. A total of 5,493 DEGs were identified based on the filtering thresholds adjusted *p*-value (adj. pval) < 0.05 and absolute log2(fold change) > 0.5. The expression data of these DEGs were subsequently presented to weighted gene coexpression network analysis (WGCNA) to identify highly similar coexpression modules. The Pearson method was utilized to construct a DEG similarity matrix. Based on the power value (*β* = 10), which affects the average degree and the independence of module connectivity (*k*), a topological overlap matrix (TOM) and an adjacent matrix (AM) were generated. When the correlation between *p*(*k*) and *k* > 0.85, the power value was regarded as ideal for constructing a scale-free topology network. Next, we established the hierarchical clustering dendrogram of the 1-TOM matrix to determine coexpression modules. Subsequently, we correlated the module Eigengenes (ME) with clinical features, including the six consensus subtypes of bladder cancer. A correlation between a module and consensus subtypes was considered significant if *R* > 0.4 or *R* ≤ 0.4.

### Gene dependency score analysis

To assess the “importance” of ERBB2 across different MIBC subtypes, we employed the gene dependency score (gDS) from a previous study (16). A lower gDS indicates that the gene is more important for cell viability. gDS ≤ 1 suggests that the loss of this gene is fatal for the cell. gDS between − 1 and 0 implies that the bereft of the gene influences cell viability but is not fatal. A gDS > 0 indicates that deletion of this gene has no effect on cell viability.

### Single-cell RNA sequence data analysis

The single-cell RNA (scRNA)-sequence dataset, GSE135337, was analyzed using the Seurat R-package. Initially, we performed cell filtering based on two criteria: a minimum of 50 detected genes per cell and a mitochondrial gene percentage of less than 5% per cell. We also excluded genes expressed in less than three cells. After quality control, we collected a total of 36,789 cells. The expression data of cells were then standardized and normalized using the Normalizedata R-function. Subsequently, we performed principal component analysis (PCA), cell clustering, and t-SNE dimensional reduction. The cells were separated into 16 clusters using FindClusters R-function. Subsequently, the FindAllMarkers R-function was employed to conduct differential expression analysis for a given cluster. The DEGs were further screened using the filtering standard of |logFC| > 1 and the adjusted *p*-value (adj. pval) < 0.05. We then annotated the cell types using the SingleR R-package. The expression distribution of GATA Binding Protein 3 (GATA3) and Forkhead Box A1 (FOXA1) was used to identify the zone where luminal cells are located, while CD44 Molecule (IN Blood Group) (CD44) and Keratin 5 (KRT5) were used to determine the zone where basal cells are probably located. Subsequently, we further analyzed the main area of ERBB expression in these luminal and basal cells.

### Mutation and copy number variation analysis

The mutation and copy number variation (CNV) situation of ERBB2 in pan-cancer were analyzed using the cBioPortal website (https://www.cbioportal.org/). To characterize the mutation condition of ERBB2 in MIBC, we obtained the mutation data from the UCSC Xena. We then analyzed the distribution of ERBB2 mutations in luminal and basal bladder cancer. Next, we observed the mRNA expression differences of ERBB2 in basal mutation type, luminal mutation type, and wild type. To explore the ERBB2 CNV across bladder cancer subtypes, we also downloaded the linear copy number data of MIBC from the UCSC Xena website. Subsequently, we calculated the Pearson correlation coefficient (PCC) between ERBB2 mRNA expression and linear copy number. To estimate the recurrent focal somatic copy number variation (SCNA), we downloaded the segmentation files of MIBC tumor samples from TCGA GDC Data Portal (https://portal.gdc.cancer.gov). We then identified the significantly recurrent focal genomic regions of MIBC, luminal MIBC, and basal MIBC using the Genomic Identification of Significant Targets in Cancer (GISTIC 2.0) algorithm, accessed through the GenePattern website (https://cloud.genepattern.org/gp/pages/index.jsf). We applied the default parameters of GISTIC 2.0 with a 0.99 confidence level and a *q*-value < 0.25. According to the GISTIC amplitude threshold, the significant focal events of the sample were classified into four types: shallow deletion, deep deletion, low-level amplification, and high-level amplification. Subsequently, a GISTIC score (G-score) for individual genes with gain or loss was generated separately in MIBC. The differences in ERBB2 mRNA expression across different types of CNV were further analyzed.

### Cell culture and treatment

Human bladder cancer cells representing luminal type (SW780, HT1376, and RT4) and basal type (5637, T24, and J82) were purchased from Procell Life Science&Technology Co. Ltd. (Wuhan, China). These cells were authenticated by short tandem repeat (STR) profiling. According to the manufacturer’s instructions, the cells were cultured in their respective media, each supplemented with fetal bovine serum and penicillin–streptomycin. All of the bladder cancer cells were cultivated in an incubator with a humidified atmosphere containing 5% CO_2_ at 37°C. The cells remained mycoplasma free throughout the culturing process.

### Immunohistochemistry

MIBC tissue biopsy specimens, formalin-fixed and paraffin-embedded (FFPE), were collected from the Second Hospital of Tianjin Medical University. Immunostaining for luminal markers (GATA3 and KRT20) and basal markers (KRT5/6 and CD44) was performed on 100 MIBC samples to identify luminal and basal bladder cancer subtypes (17). Samples that showed both luminal and basal staining or no staining were excluded. A total of 12 basal bladder cancer and 19 luminal bladder cancer tissues were finally determined. Immunostaining for ERBB2 was then performed on these 31 MIBC tissues. The specimens were first deparaffinized and rehydrated, followed by antigen retrieval in sodium citrate buffer and blocking in 3% H_2_O_2_ and acidin/biotin. Subsequently, the specimens were incubated with antibodies overnight, followed by incubation with the corresponding secondary antibody. Diaminobenzidine (DAB) was used as the chromogen, and hematoxylin counterstaining was applied. The immunohistochemistry (IHC) staining was then scored using ImageJ software. The IHC score was calculated by multiplying the staining intensity (0, negative; 1, weak; 2, moderate; 3, strong) with the proportion of immunopositive cells of interest (1, < 25%; 2, 25%–50%; 3, 50%–75% and 4, >75%).

### Protein extraction and Western blotting assay

Control and treatment cells were lyzed using a protein extraction reagent (Boster, Wuhan) supplemented with 10% PMSF (Solarbio, Beijing). The suspensions were then centrifuged, and the supernatant was collected. The concentration of the supernatant was determined using the bicinchoninic acid assay (BCA) (Solarbio, Beijing). Subsequently, the proteins were isolated and further transferred onto a polyvinylidene fluoride (PVDF) membrane (ThermoFisher, China), blocked with 5% skimmed milk powder, and incubated with primary antibodies against ERBB2, GATA3, KRT5/6, GAPDH, KRT20, CD44, STAT3, and pSTAT3 (Tyr705) at 4°C overnight. The membranes were subsequently incubated with the corresponding secondary antibodies. The blot was ultimately visualized using ECL Detection Reagents. ImageJ was utilized to compute the intensities of the detected bands.

### Drug dose–response and clone formation assay

Cells were planted in 96-well plates in quintuplicate. After 24 h, RC48 and ART were added at previously set concentrations and incubated for 48 h. The medium was then replaced with CCK8 at a ratio of 10:1 (APE×BIO, USA) for 2–4 h. Cell viability was subsequently measured based on optical density at 450 nm. The IC_50_ for each group was then calculated using GraphPad Prism 8 software. For the clone formation assay, nontreated/treated cells were cultured in 12-well plates (approximately 1,000 cells per well) for 10–20 days. The cells were then dyed with 0.05% crystal violet, and the number of colonies was quantified using ImageJ.

### Transfection

ERBB2 and STAT3 deletion cells were established by underexposing their corresponding siRNA oligonucleotides (GenePharma, Suzhou). The silencing sequences of ERBB2 and STAT3 are listed in **Supplementary Table S8**. The ERBB2 overexpression lentivirus vectors were purchased from Genechem (Shanghai, China).

### Immunofluorescence

Treated cells were plated on a glass coverslips (10 mm diameter), fixed in 4% paraformaldehyde (PFA) for 30 min, permeabilized with 0.5% TritonX-100 for 5-10 minutes, and blocked with 1% bovine serum albumin (BSA) solution for 10–15 min. Next, the cells were incubated with pSTAT3 (Tyr705) primary antibody (1:100 dilution, Affinity Biosciences, Jiangsu) at 4°C overnight and then incubated with FITC-labeled secondary antibody (1:300 dilution, Proteintech, Wuhan) for 1–2 h, finally dyed with 4′,6-diamino-2-phenylindole (DAPI, Solarbio). We then visualized the fluorescence intensity of pSTAT3 (Tyr705) under the randomly selected microscope fields (×800).

### RNA-sequencing analysis and subsequent data treatment

Total RNA from stable SW780 exposed to RC48 (25 µg/ml) and their correspondent controls was isolated. RNA-seq analysis was then performed by the Beijing Genomics Institute (BGI) using the BGIEQ-500 instrument model. The data were filtered using SOAPnuke, a filtering software developed independently by BGI. The clean reads were mapped to the reference gene sequence using Bowtie2. Gene expression values of samples were counted by RSEM. The gene read count data were later normalized and then transformed into a Log2(TPM+1) unit. Differential expression analysis was performed on the normalized data using the limma R-package. The entire RNA-seq dataset was subjected to GEO with the data series accession number GSE237789.

### Xenograft model in nude mice

This study was conducted in accordance with the Animals in Research: Reporting *In Vivo* Experiments (ARRIVE) guidelines (18). Four- to six-week-old, 24–26-g Balb/c nude mice (HFK Bioscience Co. Ltd., Beijing, China) were purchased to construct the xenograft tumor growth assay. A total of 2 * 10^6^ treated T24 cells were injected into the subcutaneous axilla to establish the xenograft tumor model. The drugs were regularly injected once the tumor in the mouse became visible. RC48 (Remegen, China) was injected via the tail vein at a dose of 10 mg/kg per mouse, once or twice a week. For ART (MedChemExpress), intraperitoneal injections were given daily at a dose of 50 mg/kg per mouse. Meanwhile, the tumor width (W) and length (L) were measured once or twice a week. The tumor volume was calculated using the formula: volume = 0.52 * L * W^2^. After approximately 3 to 4 weeks, all the mice were euthanized, the tumors were isolated, and the subsequent analyses were conducted. When the tumor diameter exceeded 0.8 cm, it was recorded as the endpoint, and the survival curve of the mice was calculated.

### Quantification and statistical analysis

All data in this study were analyzed using GraphPad Prism and R software. The unpaired *t*-test was used to assess the mean value differences between the two groups. The Chi-square test was utilized to analyze the distribution differences of matched data across various groups. The log-rank test was performed to estimate the relationship between individual variables and prognosis. The PCC was calculated to reveal the relationship between the data of two variables (^*^ *p*-value < 0.05; ^**^ *p*-value < 0.01; ^***^ *p*-value < 0.001; ^****^ *p*-value < 0.0001; ns, not significant).

## Results

### ERBB2 is negatively correlated with basal/squamous MIBC

**Figure 1:**
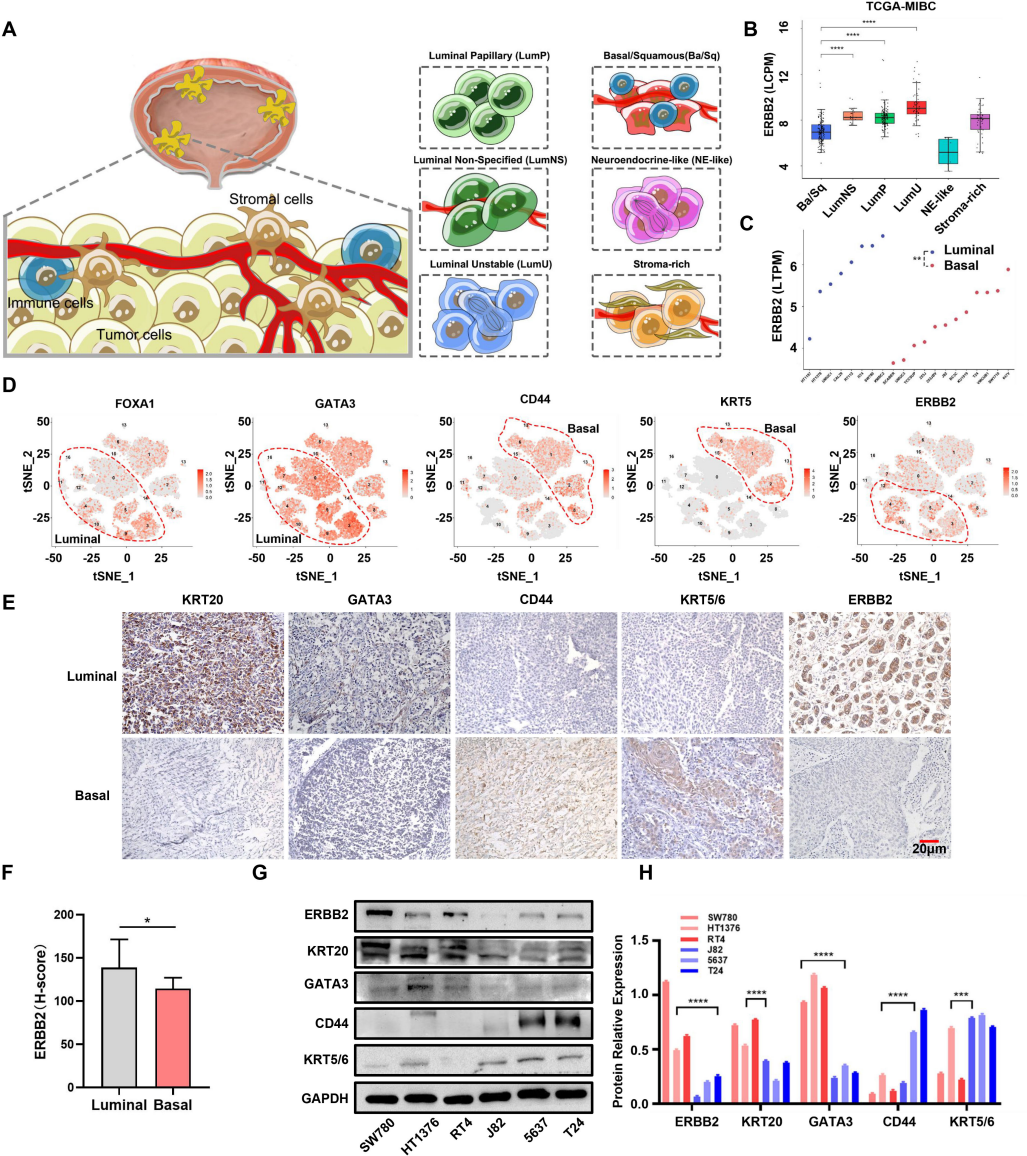
ERBB2 expression in different molecular types of MIBC. **(A)** Schematic diagram of consensus types of MIBC, including basal/squamous (Ba/Sq), luminal unstable (LumU), luminal nonspecified (LumNS), luminal papillary (LumP), neuroendocrine-like (NE-like), and stoma-rich. **(B)** ERBB2 mRNA expression in six MIBC consensus subtypes in the TCGA-MIBC dataset. **(C)** ERBB2 mRNA expression in luminal and basal cells in CCLE dataset. **(D)** The luminal and basal-type single-cell clusters were determined by their molecular markers (luminal: FOXA1 and GATA3; basal: CD44 and KRT5), and the distribution of ERBB2 mRNA expression in luminal and basal clusters is shown. **(E)** The luminal and basal bladder cancer tissues’ IHC were classified by their molecular marker (luminal: KRT20 and GATA3; basal: CD44 and KRT5/6), and ERBB2 protein expression was detected. **(F)** Quantification of ERBB2 immunohistochemistry protein levels in luminal and basal bladder cancer. **(G)** Western blot analysis showed the protein expression of ERBB2, KRT20, GATA3, CD44, and KRT5/6 in luminal (HT1376, RT4, and SW780) and basal (5637, J82, and T24) cells. **(H)** Quantitative chart of ERBB2, KRT20, GATA3, CD44, and KRT5/6 from the Western blot analysis. *, P-value < 0.05; **, P-value < 0.01; ***, P-value < 0.001; ****, P-value < 0.0001.

We used molecular classification systems to categorize MIBC patients based on mRNA expression data obtained from TCGA database. Regardless of the classification system (UNC, MDA, TCGA, or Consensus subtype), we found that patients with the basal type had worse outcomes than those with the luminal subtype (**Supplementary Figures S1A–D**). To further assess the relationship between RC48 and molecular subtypes, we first conducted a differential expression analysis comparing MIBC tissue to adjacent normal tissue. A total of 5,492 DEGs were identified (**Supplementary Figure S2A**; **Supplementary Table S1**). We then acquired the expression profile of these DEGs to perform WGCNA based on the six MIBC consensus subtypes (**Figure 1A**). A power value of *β* = 10 was chosen to construct a scale-free coexpression network (**Supplementary Figure S2B**). When the correlation between *p*(*k*) and *k* exceeded 0.85, an ideal network was further established (**Supplementary Figure S2C**). We then merged the highly similar modules using the dynamic hybrid tree-cut method with a cut-off value of 0.25 (**Supplementary Figure S2D**). Four modules were found to have a highly significant correlation with the basal/squamous subtype (yellow module: *r* = − 0.44; grey module: *r* = − 0.42; black module: *r* = 0.51; turquoise module: *r* = 0.54) (**Supplementary Figure S2E**). Intriguingly, the target of RC48, ERBB2, was located in the yellow module and showed a negative correlation with basal/squamous subtype (*R* = − 0.46737, *p*-value = 2.28*e*−23) (**Supplementary Figure S2F**; **Supplementary Table S2**). Therefore, we speculated that basal bladder cancer exhibits lower ERBB2 expression and is less sensitive to RC48.

### ERBB2 shows lower expression in basal/squamous MIBC

To validate our speculation, we first analyzed ERBB2 mRNA and protein levels across the six consensus subtypes in TCGA-MIBC dataset. ERBB2 was highly expressed in three luminal subtypes, while its expression was low in the basal/squamous subtype (**Figure 1B**; **Supplementary Figure S3A**). We then utilized five other subtype classification systems to categorize the tumor tissues based on mRNA expression profiles from TCGA-MIBC and other GEO datasets (including GSE13507, GSE32894, and GSE48075). The results showed the same trends (**Supplementary Figures S3B–E**). In addition, ERBB2 was also upregulated in luminal cells (including HT1197, HT1376, UMUC1, CAL29, RT112, and RT4) compared to basal cells (including SCABER, UMUC3, TCCSUP, 253J, 253JBV, J82, BC3C, KU1919, T24, VMCUB1, SW1710, and 647V) (*p*-value < 0.01) (**Figure 1C**). We further conducted cluster analysis using the bladder cancer single-cell RNA-sequencing dataset GSE135337. Applying the luminal (including FOXA1 and GATA3) and basal (including CD44 and KRT5) markers, we observed the expression distribution of these genes in epithelial cells. Interestingly, ERBB2 was mainly enriched in the cell clusters expressing GATA3 and FOXA1 (**Supplementary Figure S3F**; **Figure 1D**). In five bladder cancer datasets (TCGA-MIBC, GSE13507, GSE48075, GSE32894, and GSE48276), ERBB2 was highly positively correlated with GATA3 and FOXA1 but negatively correlated with CD44 and KRT5, suggesting that ERBB2 is a luminal marker to some extent (**Supplementary Figures S4A–E**). We then performed immunohistochemistry using luminal (KRT20 and GATA3) and basal (CD44 and KRT5/6) markers, excluding the mixed types. A total of 12 basal and 19 luminal MIBC samples were determined. We found that ERBB2 was highly expressed in luminal bladder cancer (*p*-value < 0.01) (**Figures 1E, F**). Moreover, ERBB2 was upregulated in luminal cells compared to basal cells (**Figures 1G, H**; **Supplementary Figure S5A**). Notably, luminal and basal cells exhibited different growth morphologies: cohesive clusters of epithelial cells in luminal cells, and mesenchymal and squamous differentiated morphology in basal cells (**Supplementary Figure S5B**). In summary, ERBB2 was highly enriched in luminal bladder cancer and expressed at lower levels in basal bladder cancer.

### Alterations in ERBB2 expression in luminal and basal bladder cancer are associated with CNV and somatic mutations

To excavate the cause of the expression alterations of ERBB2 in luminal and basal bladder cancer, we first focused on CNV and somatic mutations. We observed frequent CNV and mutations of ERBB2 in bladder cancer (**Supplementary Figure S6A**). The mutation frequency of ERBB2 in basal and luminal bladder cancer did not differ significantly, with missense mutation being the primary mutation type (**Supplementary Figures S6B, C**). Notably, luminal mutation samples showed a significant upregulation of ERBB2 compared to basal mutation and wild-type samples (**Supplementary Figure S6D**). In addition, we observed an upregulation of ERBB2 linear copy number in luminal bladder cancer (**Supplementary Figure S7A**). ERBB2 mRNA expression was highly correlated with its linear copy number (**Supplementary Figure S7B**). We further analyzed SNP array profiles from TCGA to identify recurrent focal peaks (*q*-value < 0.25) using GISTIC 2.0 for bladder cancer. We found that the recurrent focal peaks encompassed chromosome sites where ERBB2 is located (**Supplementary Figure S7C**). Among these, basal bladder cancer showed a lower peak, while luminal bladder cancer exhibited a higher peak. The G-score considers both the frequency of occurrence across samples and the amplitude of the aberration. We found that the chromosomal locus containing ERBB2 exhibited a lower G-score in basal bladder cancer and a higher G-score in luminal bladder cancer (**Supplementary Figure S7D**). Moreover, we observed a higher rate of ERBB2 amplification in luminal bladder cancer (**Supplementary Figure S7E**). Compared to the diploid samples, amplification (both low and high level) that occurred in luminal samples was associated with upregulated ERBB2 mRNA expression, whereas basal samples showed no significant change in mRNA levels (**Supplementary Figure S7F**). In summary, the copy number variant of ERBB2 was more frequent and had a greater influence on ERBB2 mRNA expression levels in luminal bladder cancer compared to basal bladder cancer.

### Luminal cells show higher sensitivity to RC48

**Figure 2:**
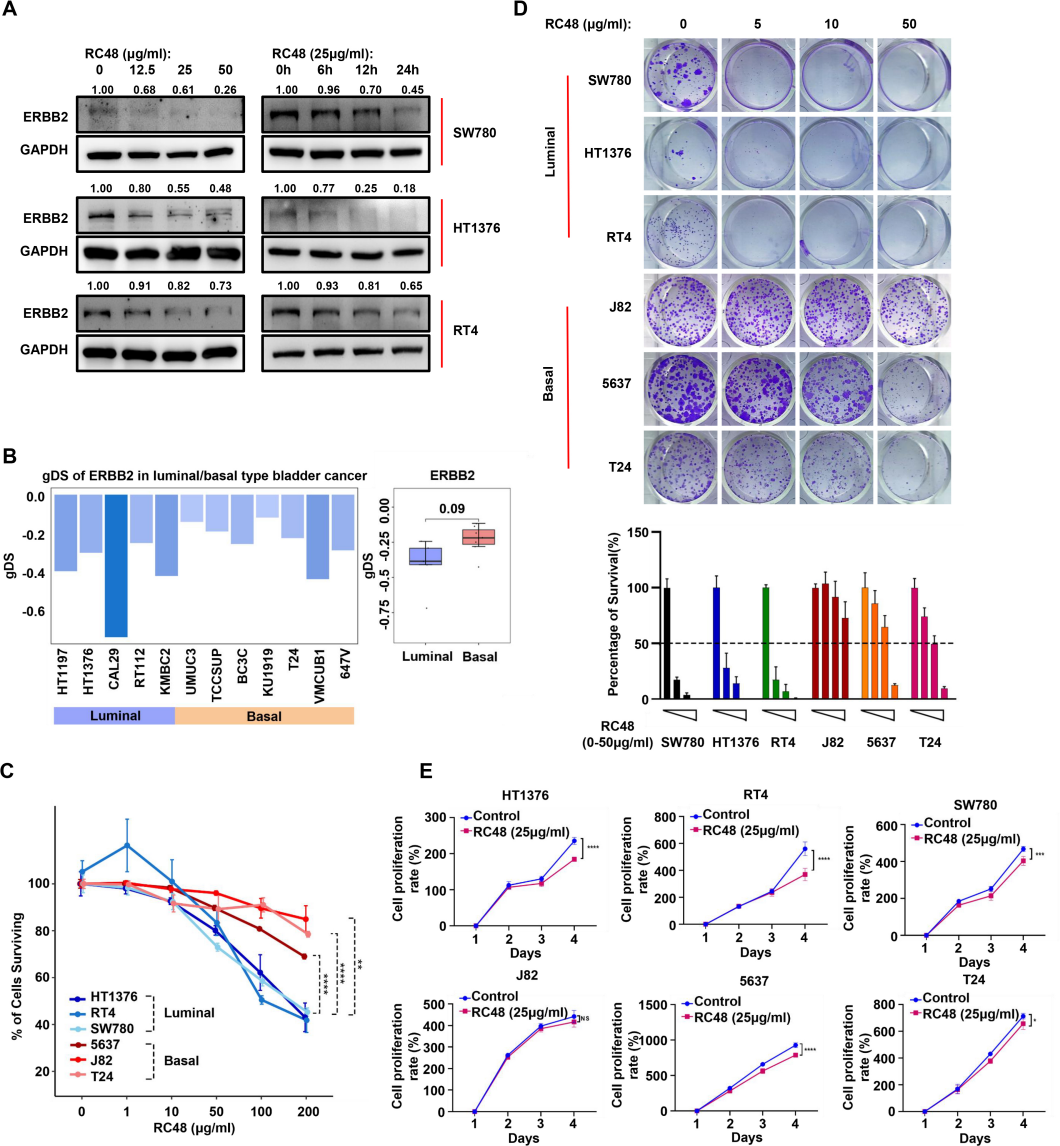
Luminal cells show higher sensitivity to RC48. **(A)** The Western blot shows ERBB2 protein expression levels in luminal cells (SW780, HT1376, and RT4) after exposure to RC48 at different concentrations and times. **(B)** The barplot shows the gDS in MIBC cells representing luminal and basal cells. **(C)** The percentage of surviving cells after exposing different concentrations of RC48 in three luminal (SW780, RT4, and HT1376) and three basal (J82, T24, and 5637) cells. **(D)** Colon formation assay reveals the cell growth after exposing different concentrations of RC48 in luminal and basal cells. The percentage of survival cells was quantified. **(E)** Cell viability of three luminal and three basal cells after exposing RC48 (25 μg/ml) at different times. *, P-value < 0.05; **, P-value < 0.01; ***, P-value < 0.001; ****, P-value < 0.0001; ns, not significant.

Due to the heterogeneous expression and epigenetic alterations of ERBB2 in luminal and basal bladder cancer, we speculated that these two subtypes might exhibit different sensitivities to RC48. In three luminal cells, RC48 inhibited ERBB2 expression in a time- and dose-dependent manner (**Figure 2A**). We further used gDS to explore the effect of ERBB2 deletion in luminal cells (HT1197, HT1376, CAL29, RT112, and KMBC2) and basal cells (UMUC3, TCCSUP, BC3C, KU1919, T24, VMCUB1, and 647V). A lower gDS indicates a higher importance of genes in tumor cells (16). A gene with a gDS ≤ 1 suggests that its deletion seriously influences cell viability, while − 1 < gDS < 0 indicates that gene deletion affects cell viability to some extent. We found that the gDS of ERBB2 was less than 0 all these bladder cells, with lower scores in luminal cells compared to basal cells (unpaired *t*-test: *p*-value = 0.09) (**Figure 2B**; **Supplementary Table S3**). The results indicated that ERBB2 deletion might have a far more severe effect on the viability of luminal cells compared to basal cells. After adding different concentrations of RC48 to luminal (SW780, RT4, HT1376) and basal (J82, T24, 5637) bladder cancer cells, we found that RC48 could inhibit the viability of these cells in a dose-dependent manner. Among these, the effect was more serious and significant in luminal cells (**Figure 2C**). In addition, RC48 significantly inhibited the growth of luminal cells (IC_50_: HT1376 = 158.3 μg/ml, SW780 = 155.7 μg/ml, RT4 = 132.2 μg/ml) but showed a weaker function against the basal cells (IC_50_: 5637 > 200 μg/ml, T24 > 200 μg/ml, J82 > 200 μg/ml) (**Figure 2D**). The fixed concentration of RC48 (25 µg/ml) showed a more significant effect on luminal cells than basal cells over time (**Figure 2E**). In summary, luminal cells were more sensitive to RC48 than basal ones.

### The function of RC48 against tumor cells depends on ERBB2 expression

**Figure 3:**
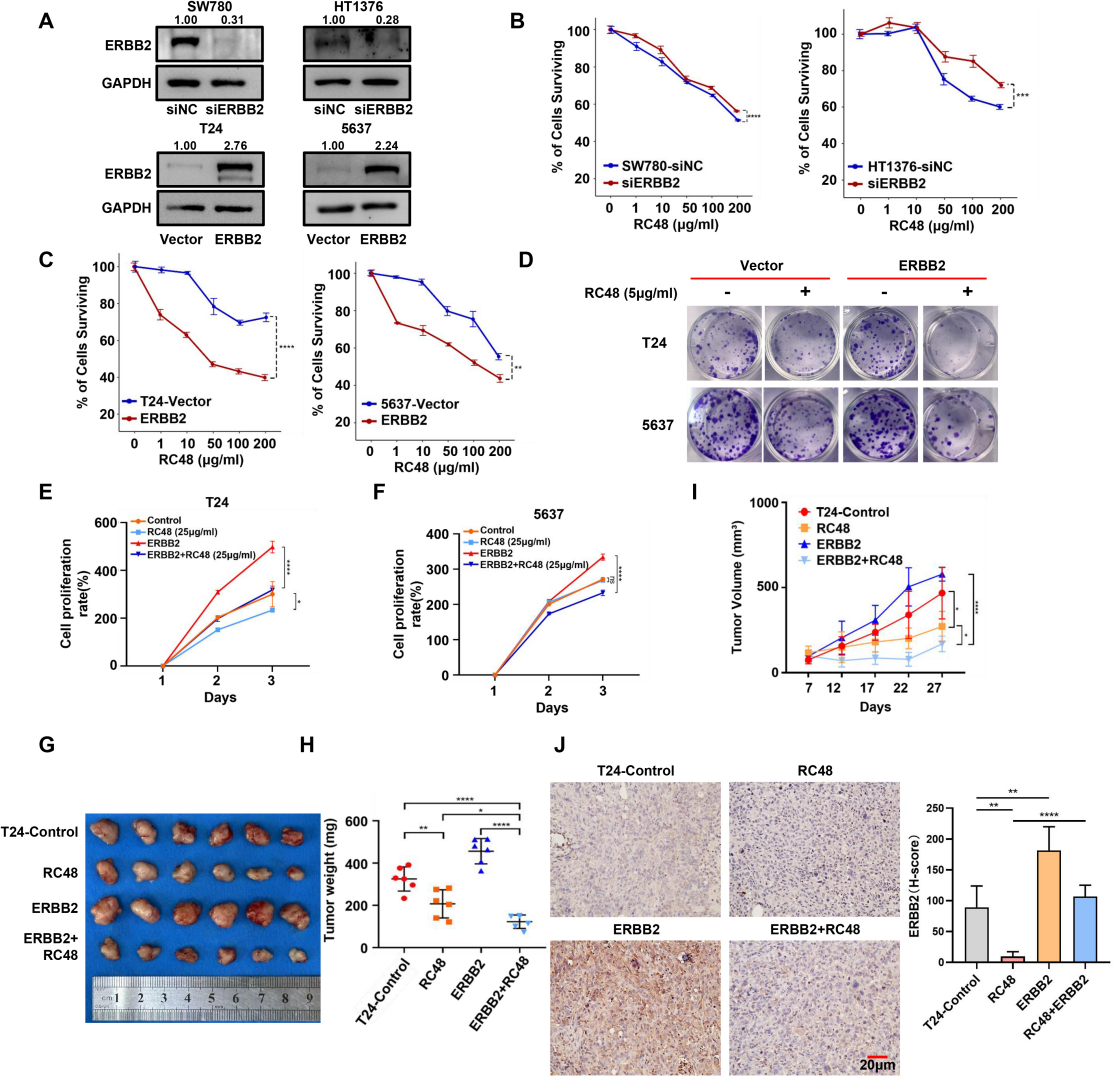
ERBB2 expression mediates the sensitivity of luminal and basal cells to RC48. **(A)** Western blot analysis validated the ERBB2 expression in ERBB2-silenced luminal cells (SW780 and HT1376) and ERBB2-overexpressing basal (T24 and 5637) cells. **(B**, **C)** The percentage of surviving cells after exposure to different concentrations of RC48 in ERBB2-silenced luminal cells (SW780 and HT1376)/or ERBB2-overexpressing basal cells (T24 and 5637). **(D)** Colon formation assay demonstrates cell growth after exposure to RC48 (5 μg/ml) in ERBB2-overexpressing basal cells. **(E**, **F)** Cell viability of the ERBB2-overexpressing and control basal cells after exposure to RC48 (25 μg/ml). **(G)** Tumors with different treatments were shown. **(H)** Tumor weight was measured after 4 weeks. Data are presented as the mean ± SD. **(I)** Tumor volume was measured one to two times weekly once the tumor became visible. Data are presented as the mean ± SD. **(J)** IHC revealed ERBB2 expression in tumor samples from mice receiving different treatments. *, P-value < 0.05; **, P-value < 0.01; ***, P-value < 0.001; ****, P-value < 0.0001.

To explore whether ERBB2 expression mediates the sensitivity of cells to RC48, we further constructed ERBB2-silenced and overexpressed cells (**Figure 3A**). We found that ERBB2-silenced luminal cells (SW780 and HT1376) exhibited reduced sensitivity to RC48 (**Figure 3B**). In contrast, ERBB2-overexpressing basal cells (T24 and 5637) showed increased sensitivity to RC48 (**Figures 3C, D**). After exposure to RC48 for 48 h, the cell proliferation rate was inhibited approximately 1.2–1.5 times in T24/5637-Control cells. Meanwhile, T24/5637-ERBB2-overexpressed cells inhibited cell proliferation approximately two times (**Figures 3E, F**). We further constructed a xenograft tumor model in mice using control and ERBB2 overexpression T24 cells. After regular RC48 treatment, we found a higher treatment efficacy of drugs in the group of ERBB2 overexpressed group (**Supplementary Figure S8**; **Figure 3G**). The tumor growth was significantly inhibited in the ERBB2 overexpressed group after treating the drug (**Figures 3H, I**). In addition, for the control group with RC48 treatment, ERBB2 expression was not significantly inhibited than without the RC48 treatment control group. This might be a consequence of its low expression of ERBB2. In the ERBB2 overexpressing group, ERBB2 expression was significantly inhibited after treatment with RC48 (**Figure 3J**). Overall, these results indicated that RC48’s efficacy against bladder cancer cells depends on ERBB2 expression.

### RC48 stimulates the activation of JAK/STAT3 pathways

**Figure 4:**
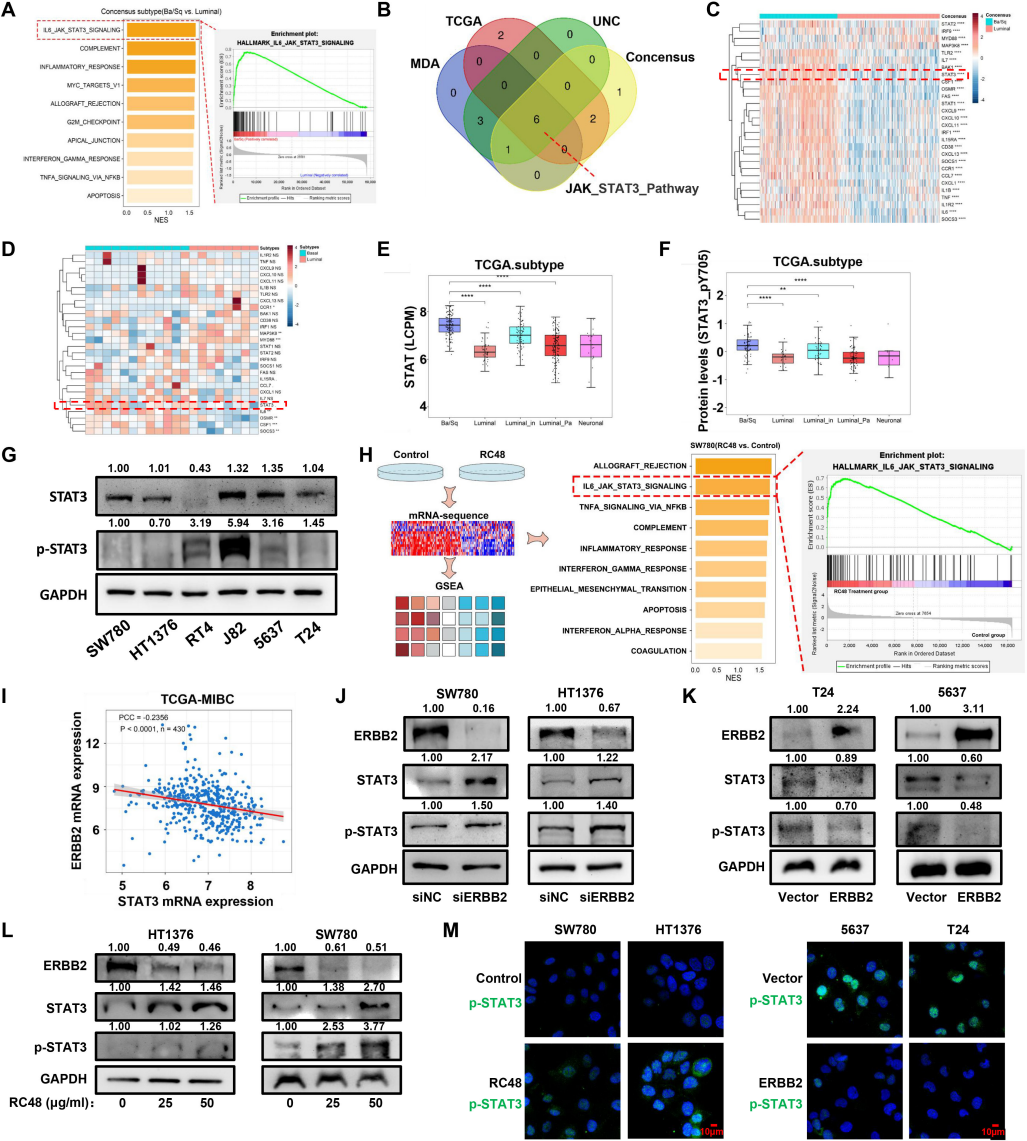
The STAT3 pathway is compensatively activated in basal cells. **(A)** The diagram shows the GESA result based on the DEGs between basal/squamous and the other three luminal consensus subtypes. **(B)** The Venn diagram shows the common altered pathways from the GSEA results based on the DEGs determined between basal and luminal types of four molecular classification systems. **(C, D)** The heatmap shows the expression of genes positively correlated with STAT3 pathways in basal and luminal bladder cancer tissues and cell lines. **(E)** STAT3 mRNA expression levels in consensus types of TCGA-MIBC. **(F)** STAT3 mRNA expression levels in luminal and basal cells. **(G)** STAT3 protein levels in luminal and basal cells were detected by Western blot analysis. **(H)** The diagram shows the GSEA results based on the DEGs between RC48-exposed SW780 and control. The top 10 activated pathways are shown. **(I)** The diagram shows the correlation between ERBB2 and STAT3 in mRNA levels, with the PCC used for analysis. **(J, K)** In ERBB2-silenced luminal cells and ERBB2-overexpressing basal cells, Western blot analysis was performed to measure the expression changes of ERBB2, STAT3, and pSTAT3 (Tyr705). **(L)** After exposure to RC48 (HT1376 and SW780), the proteins ERBB2, STAT3, and pSTAT3 (Tyr705) were detected by Western blot analysis. **(M)** Control and RC48-exposed SW780 and HT1376 cells, or ERBB2-overexpressing T24 and 5637 cells, were fixed and incubated with antibodies against pSTAT3 (Tyr705) for immunofluorescence analysis. *, P-value < 0.05; **, P-value < 0.01; ***, P-value < 0.001; ****, P-value < 0.0001.

To increase the sensitivity of basal cells to RC48, it is necessary to explore other drugs for combination therapy (19). We first collected the mRNA expression profile from TCGA-MIBC dataset and then conducted a differential expression analysis between basal/squamous and three other luminal subtypes (including LumNS, LumP, and LumU) of bladder cancer. The DEGs were subsequently analyzed using GSEA software. The top 10 hallmark pathways were finally identified and ranked by the normalized enrichment score (NES) (**Figure 4A**). Among these, JAK/STAT3 pathways showed the highest NES. We further analyzed basal and luminal types determined by other classification systems (**Supplementary Figure S9A**). We found the recurrent high enrichment score of the JAK/STAT3 pathway in basal bladder cancer (**Figure 4B**; **Supplementary Table S4**). These results revealed that the JAK/STAT3 pathway highly activated basal bladder cancer. The previous study provided a PROGENy algorithm showing a common core of pathway-responsive genes by leveraging a large-scale compendium of available perturbation experiments (20). The weight > 0 of the gene indicates that the activation of the pathways accompanies gene expression. We selected the genes with a weight > 1 of JAK/STAT3 pathways and observed the mRNA expression in basal and luminal bladder cancer (**Supplementary Table S5**). We observed that most genes, including STAT3, STAT1, STAT2, and IL6, exhibited higher expression in the basal subtype (**Figure 4C**). In luminal and basal cells, we found a trend of upregulation for STAT3 and IL6 (**Figure 4D**). In TCGA, GEO, and CCLE datasets, we also observed that the expression of STAT3 and pSTAT3 is lower in luminal bladder cancer than in basal bladder cancer (**Figures 4E, F**; **Supplementary Figures S9B–D**, **S10A–D**). STAT3 represented a negative relation with luminal markers, such as KRT20 and GATA3, but showed a positive relation with basal markers, including KRT5 and CD44 (**Supplementary Figures S11A–E**). Western blot analysis revealed that STAT3 and pSTAT3 are highly enriched in basal cells (J82, 5637, and T24) compared to luminal cells (SW780, HT1376, and RT4) (**Figure 4G**). A previous study also uncovered that pSTAT3 significantly increases in infiltrating basal bladder cancer compared to luminal bladder cancer (21). To further determine whether the JAK/STAT3 pathway occurred as a compensatory activation based on the downregulation of ERBB2 in basal bladder cancer, we performed RNA-sequencing to explore this speculation. After exposing RC48, we found that the JAK/STAT3 pathway showed a higher activation (**Figure 4H**; **Supplementary Tables S6**, **S7**). Genes (including IRF9, MAP3K8, CSF1, CXCL1, IL1B, CXCL10, MYD88, STAT3, STAT3, TNF, SOCS1, and SOCS3) positively correlate with the upregulation of JAK/STAT3 pathways in RC48-exposed SW780 (**Supplementary Figure S12A**). In addition, in multiple bladder cancer datasets (including TCGA-BLCA, GSE13507, GSE48075, and GSE32894), we observed a negative correlation between STAT3 and ERBB2 in mRNA levels (**Figure 4I**; **Supplementary Figure S12B**). We further silenced and overexpressed ERBB2 in luminal and basal cells. STAT3 and pSTAT3 (Tyr705) were upregulated under ERBB2 silencing and downregulated under ERBB2 overexpression (**Figures 4J, K**). Upon RC48 exposure, we also found that STAT3/pSTAT3 (Tyr705) were upregulated in HT1376 and SW780 (**Figure 4L**). Furthermore, pSTAT3 (Tyr705) was highly expressed in the nucleus after exposure in SW780 and HT1376 but showed a lower nuclear expression after ERBB2 overexpression in 5637 and T24 cells (**Figure 4M**). Moreover, in TCGA-MIBC and other GEO datasets, we observed that the patients with high STAT3 expression or high STAT3-pY705 expression showed worse clinical outcomes (**Supplementary Figures S13A–D**). Additionally, STAT3 expression was correlated with cancer progression (**Supplementary Figure S13E**). These results indicate that a compensatory mechanism for STAT3 activation exists following RC48 exposure, suggesting that targeting STAT3 could be a feasible strategy for combining RC48 against basal bladder cancer.

### STAT3 inhibition acts synergistically with ERBB2 inhibition in basal cells

**Figure 5:**
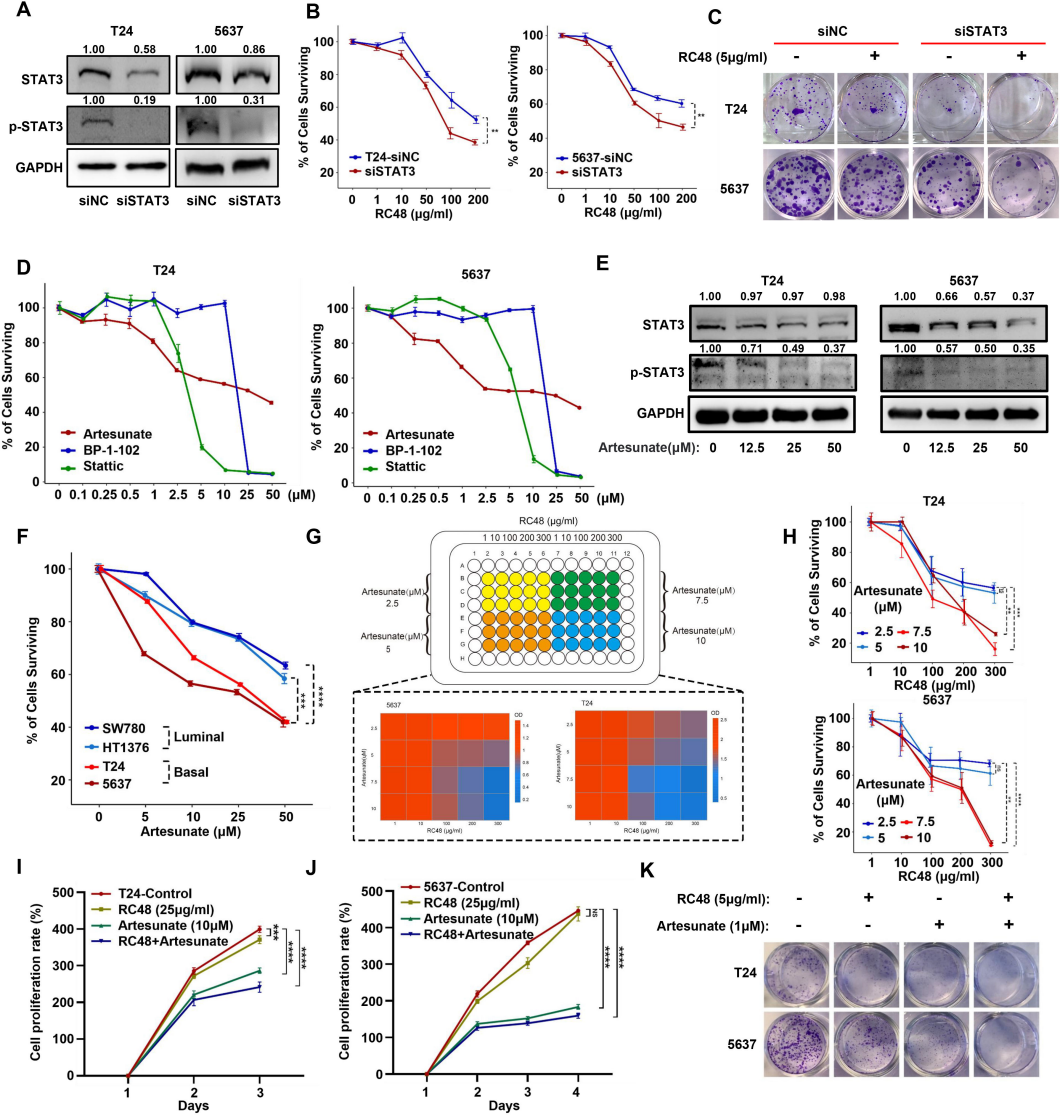
STAT3 silencing and inhibition can increase the sensitivity of basal cells to RC48. **(A)** Western blot analysis validated the STAT3/pSTAT3 (Tyr705) protein expression in STAT3-silenced basal cells (T24 and 5637). **(B)** The percentage of surviving cells after exposure to RC48 (5 μg/ml) in STAT3-silenced basal cells and their corresponding control cells. **(C)** Colony formation assay revealed cell growth after exposure to RC48 (5 μg/ml) in STAT3-silenced cells and their corresponding control cells. **(D)** Dose–response assay of three STAT3 inhibitors in T24 and 5637 cells. **(E)** Western blot analysis revealed STAT3 and pSTAT3 (Tyr705) expression after exposure to different concentrations of ART. **(F)** The percentage of surviving cells after exposure to different concentrations of ART in basal (T24 and 5637) and luminal (SW780 and HT1376) bladder cancer cells. **(G, H)** Cell viability was assessed by combining different concentrations of RC48 and ART in T24 and 5637 cells. **(I, J)** The CCK-8 proliferation assay was performed on T24 and 5637 cells exposed to saline, a single drug (RC48 or ART), or a combination of both drugs. **(K)** The colony formation assay was conducted on T24 and 5637 cells exposed to saline, a single drug (RC48 or ART), or a combination of both drugs. **, P-value < 0.01; ***, P-value < 0.001; ****, P-value < 0.0001; ns, not significant.

To investigate whether inhibition of STAT3 could improve the anticancer effect of RC48, we first silenced STAT3 in T24 and 5637 cells (**Figure 5A**). STAT3 silencing increases the sensitivity of RC48 against basal cells (**Figure 5B**). Under STAT3-silencing conditions, RC48 significantly inhibited cell growth (**Figure 5C**). Previous studies have revealed that ART, BP-1-102, and Stattic can inhibit the activity of JAK/STAT3 signaling (22–24). We further utilized these inhibitors to explore their effects in combination with RC48 against basal cells. After exposing cells to different drug concentrations, we found that ART could dose-dependently inhibit cell viability effectively. In contrasts, Stattic and BP-1-102 demonstrated significant single-agent cytotoxicity, reducing their potential utility for further combination therapy (**Figure 5D**). Therefore, we selected ART for further investigation. After treating T24 and 5637 cells with different concentrations of ART, STAT3 and pSTAT3 (Tyr705) expression were significantly inhibited in a dose-dependent manner (**Figure 5E**). Moreover, we observed that ART could significantly inhibited the viability and growth of basal cells more than luminal cells (**Figure 5F**; **Supplementary Figures S14A, B**). Combining ART with RC48 significantly inhibited the viability and growth of basal cells (**Figures 5G–K**). Furthermore, STAT3/pSTAT3 (Tyr705) activation induced by RC48 in both luminal and basal cells was reversed by ART (**Supplementary Figures S14C, D**). Utilizing other agents (including Stattic and BP-1-102) with an inhibition effect on STAT3, we also observed increased anticancer efficacy combined with RC48 (**Supplementary Figures S15A–F**).

### A combination of STAT3 and ERBB2 inhibition reduces basal bladder cancer growth *in vivo*

**Figure 6:**
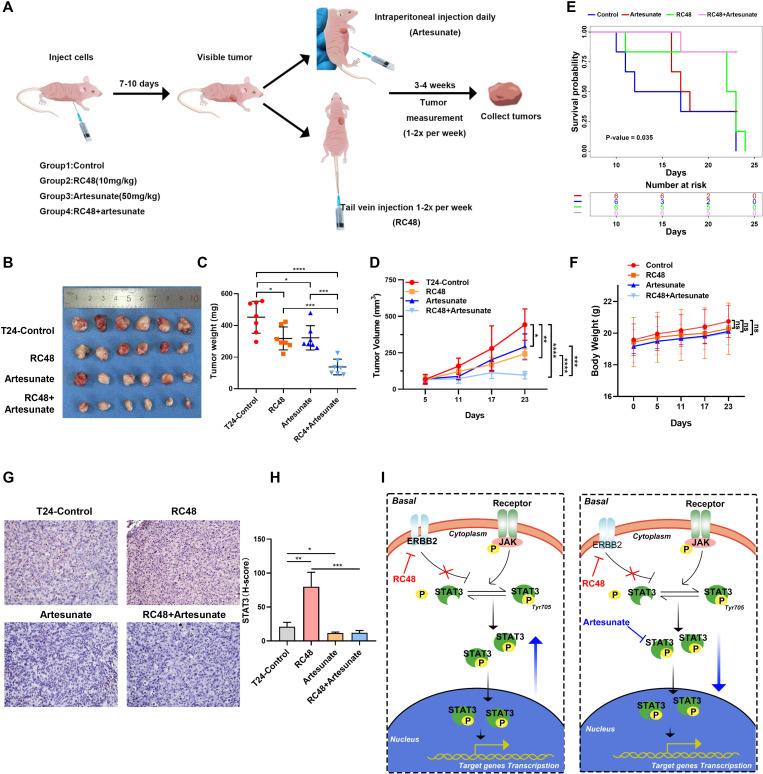
The combination of ART and RC48 blocks tumor growth in the xenograft model of nude mice. **(A)** Schematic diagram of the treatment paradigm. **(B)** Tumors with different treatments are shown. **(C)** Tumor weight was measured after 4 weeks. Data are presented as the mean ± SD. **(D)** Tumor volume was measured one to two times per week when the tumor became visible. Data are presented as the mean ± SD. **(E)** The Kaplan–Meier survival curve shows the prognostic differences among the control, RC48, artesunate, and RC48+artesunate groups. **(F)** The diagram shows changes in mouse body weight. **(G)** IHC shows the STAT3 expression in tumor samples from mice with different treatments. **(H)** Diagram of STAT3 pathways after treatment with RC48 in luminal and basal cells. **(I)** A combination of STAT3 and ERBB2 inhibition in vivo and mechanism diagram. *, P-value < 0.05; **, P-value < 0.01; ***, P-value < 0.001; ****, P-value < 0.0001; ns, not significant.

We further examined the effect of combining ART and RC48 for T24 cell xenograft tumors planted in mice (**Figure 6A**). The detailed experimental procedure is shown in **Figure 6A**. Compared to the control group, RC48 with 10 mg/kg and ART with 50 mg/kg slightly reduced tumor size and weight. However, the combination of both drugs resulted in a significant reduction in tumor size and weight compared to both the control group and the groups treated with a single drug (**Figures 6B–D**). The control group shows the worst prognosis, while the combination treatment group exhibited the best prognosis (**Figure 6E**). The body weight of mice showed no significant changes across the four groups during the duration of the treatment (**Figure 6F**). Immunohistochemical results indicated that RC48 could upregulate STAT3 expression. The upregulation of STAT3 could be reversed by ART (**Figures 6G, H**). All in all, the combination of inhibitors targeting STAT3 and ERBB2 acted synergistically to enhance the therapeutic efficacy of mice xenograft tumors. Furthermore, ART increased the sensitivity of RC48 against basal bladder cancer (**Figure 6I**).

## Discussion

For locally advanced and metastatic bladder cancer, current treatment options are limited. Due to the heterogeneity of BLCA, patients receiving the same therapy often exhibit distinct clinical outcomes. Therefore, exploring the individualized treatment of different types of patients is necessary. Advances in sequencing efforts and large-scale gene expression analyses have enabled clinicians to classify BLCA into different molecular subtypes (25). Since 2014, multiple molecular classifications of MIBC have been established, including Baylor types (26), UNC types (27), MDA types (28), TCGA types (29), CIT-curie types (30), and Lund types (31). In recent years, consensus subtypes of MIBC have been determined (12), providing guidance for clinicians and researchers to discover potential biomarkers or optimal drugs based on the different subgroups of the patients (13, 14). In our study, we observed that patients exhibited different clinical outcomes across consensus subtypes, sparking our interest in investigating the underlying mechanisms and potential drugs based on different molecule subtypes.

ERBB2 has been reported to be highly enriched in bladder cancer and associated with poor clinical outcomes (32, 33), suggesting that it might be a promising therapeutic target. In February 2022, ERBB2-targeting RC48 was admitted by China’s National Medical Products Administration for the treatment of patients with locally advanced or metastatic urothelial cancer exhibiting high ERBB2 expression who had previously failed platinum-based chemotherapy (34). For these patients, RC48-ADC represented a superior benefit/risk profile, increased ERBB2 selectivity, and good tolerability. Notably, in patients with liver metastasis, RC48 exhibited better clinical activity and disease control. However, patients with bladder cancer showed different response rates to RC48 (11). In addition, the efficacy of RC48 in treating different molecular subtypes of bladder cancer remains unclear. Nevertheless, evidence suggests that ERBB2 exhibits different levels of enrichment across different bladder cancer subtypes (12). Our study found that ERBB2 expression was higher in luminal bladder cancer and negatively correlated with basal bladder cancer. Furthermore, ERBB2 silencing resulted in lower gDS in luminal bladder cancer. These results give us confidence in exploring the therapeutic effect of ERBB2-targeting RC48 based on molecular subtypes. In line with this, RC48 demonstrates a more significant anticancer effect against luminal cells than basal cells. Downregulation of target surface protein expression serve as a resistance mechanism to ADC treatment. In metastatic breast cancer, downregulation of ERBB2 contributes to resistance to the ERBB2-targeted trastuzumab emtansine (35). Consistent with this, ERBB2 expression mediates the sensitivity of bladder cancer cells to RC48. Restoring ERBB2 expression could enhance the treatment efficacy of RC48 against basal cells. However, given that ERBB2 overexpression is associated with disease progression in bladder cancer (33, 36), supplementing ERBB2 expression to treat basal bladder cancer is clearly not a viable approach. Therefore, exploring the potential mechanism of resistance to RC48 could provide an effective therapeutic strategy for treating basal bladder cancer.

Through GSEA analysis and RNA sequencing, we found that the JAK/STAT3 pathway is activated in basal bladder cancer and in RC48-exposed SW780 cells. A previous study showed that STAT3-mediated feedback activation, caused by drug treatments such as EGFR inhibitors, contributes to resistance, acting as a cell-protective feedback mechanism (37). Disrupting or blocking STAT3 feedback could enhance drug efficacy in many oncogene-addicted cells. The relationship between the JAK/STAT3 pathway and drug resistance has been frequently reported (38, 39). In bladder cancer, cells with doxorubicin (DOX) and cisplatin (CIS) chemoresistance, accompanied by STAT3 activation, showed that the resistance could be reversed by a STAT3 inhibitor (40). Therefore, we speculated that STAT3 inhibitors also could also enhance RC48 efficacy against basal bladder cancer. Through STAT3 silencing and a combination of a STAT3 inhibitor and RC48, we observed encouraging efficacy in eliminating tumors *in vitro* and *in vivo*. Of note, we selected ART as the combination drug with RC48 rather than BP-1-102 and Stattic. ART demonstrated dose-dependent inhibition of basal bladder cancer cell viability, indicating that it is a safe and controllable drug. From the beginning, artemisinin (ARS) and its derivatives, including ART, have been used as standard treatments for malaria worldwide (41, 42). With the development of research, accumulated research revealed that ARS and its derivatives could effectively inhibit tumor cells by various pathways, such as oxidative stress, DNA damage, and repair, various programmed-cell death, and signal transducers (JAK/STAT3, MYC/MAX, NF-κB, etc.) (42). Multiple clinical trials have reported that ART (ART) treatments are well-tolerated, with no significant increase in toxicity and only minor side effects (43–47). For instance, a clinical trial involving advanced non-small cell lung cancer showed that a combination of ART and cisplatin had a strong inhibitory effect, with ART not causing a significant increase in toxicity (43). Another study involving 23 colorectal carcinoma patients treated with ART demonstrated well-tolerated tumor control (45). A trial involving 23 metastatic breast cancer patients treated with ART reported nonsevere ART-related adverse events affecting the auditory system (46, 47). Therefore, considering ARS and ART as a natural combination therapy may be more effective against tumors (42). Our research well revealed the inhibition effects of ART-combined RC48 against basal bladder cancer both *in vivo* and *in vitro*. Therefore, these combination strategies could be considered for treating this type of bladder cancer. Of note, consistent with previous studies (17), we found that many patients exhibit a mixture type of luminal and basal characteristics, suggesting that most MIBC patients could benefit from a combination approach (17). Furthermore, due to the low toxicity and good tolerability of ART, regular treatment with ART could be considered for bladder cancer patients. However, this combination strategy still needs to be validated through further clinical trials, including assessments of anticancer efficacy and safety. A proportion of patients treated with ART may still experience reversible hepatotoxicity.

## Supplementary material

Supplementary Figure 1 Kaplan-Meier survival curve reveals the survival probability of the MIBC types based on different type classification systems, UNC subtype **(A)**, MDA subtype **(B)**, Consensus subtype **(C)**, and TCGA subtype **(D)**.

Supplementary Figure 2 Bioinformatics analyzed the potential correlation between MIBC subtypes and RC48. **(A)** The volcano diagram showed DEGs between MIBC and normal tissues. **(B)** The diagram showed the scale-free analysis and meant connectivity for different soft thresholds (power) in the TCGA-MIBC dataset. **(C)** The β = 10 was determined, and the scale-free topology was checked by assessing the correlation between p(k) and K. **(D)** The dendrogram of the DEGs between MIBC and normal bladder tissue. **(E)** Correlation between clinical status and module eigengenes (Six consensus subtypes). **(F)** ERBB2 was located in the yellow module and showed a negative correlation with the basal/squamous MIBC.

Supplementary Figure 3 ERBB2 showed heterogenous expression in MIBC across molecular subtypes. **(A)** ERBB2 protein expression in MIBC consensus types in TCGA-MIBC dataset. The sample of the NE-like type was not detected. **(B-E)** ERBB2 mRNA expression in different molecular types based on the classification systems in TCGA-MIBC, GSE13507, GSE32894, and GSE48075 datasets. **(F)** The bubble plot indicated the average expression of ERBB2 in different single-cell clusters.

Supplementary Figure 4 Correlation between ERBB2 and other markers representing luminal (GATA3 and FOXA1) and basal (CD44 and KRT5) MIBC in TCGA-MIBC **(A)**, GSE13507 **(B)**, GSE32894 **(C)**, GSE48075 **(D)**, and GSE48276 **(E)**.

Supplementary Figure 5 Growth morphoglogy of luminal cells (HT1376, RT4, and SW780) and basal cells (5637, J82, and T24).

Supplementary Figure 6 Mutation characterization of ERBB2 in MIBC. **(A)** Epigenetic alteration frequency of ERBB2 across 33 cancer types. **(B)** Mutation rate distribution in basal and luminal bladder cancer. **(C)** The percentage of mutation type in basal and luminal bladder cancer. **(D)** ERBB2 mRNA expression in basal mutation type, luminal mutation type, and wild type of bladder cancer.

Supplementary Figure 7 Copy number variation characterization of ERBB2 in MIBC. **(A)** Linear copy number of ERBB2 in different types of bladder cancer. **(B)** Correlation between ERBB2 mRNA and linear copy number. **(C)** The recurrent focal peaks for TCGA-BLCA, basal BLCA, and luminal BLCA. The location of ERBB2 was labeled. **(D)** G-score for TCGA-BLCA, basal BLCA, and luminal BLCA. The location of ERBB2 was labeled. **(E)** Distribution of different types of CNV in basal and luminal bladder cancer. **(F)** ERBB2 mRNA expression in different types of CNV.

Supplementary Figure 8 Tumor-bearing mice with different treatments was shown.

Supplementary Figure 9 Potential molecular mechanism and STAT3/pSTAT3 expression between basal and luminal bladder cancer. **(A)** The DEGs between basal and luminal bladder cancer were subjected to GSEA, and the top 10 activated pathways were shown. **(B)** STAT3 mRNA expression in different molecular subtypes of bladder cancer based on different classification systems in the TCGA-MIBC dataset. **(C)** pSTAT3 (Tyr705) expression in different molecular subtypes of bladder cancer based on different classification systems in the TCGA-MIBC dataset. **(D)** STAT3 mRNA expression in luminal and basal cells in the CCLE dataset.

Supplementary Figure 10 STAT3 mRNA expression in different molecular types of bladder cancer based on different classification systems in GSE13507 **(A)**, GSE32894 **(B)**, GSE48075 **(C)**, and GSE48276 **(D)**.

Supplementary Figure 11 Correlation between STAT3 and markers representing luminal (KRT20 and GATA3) and basal (KRT5 and CD44) types in TCGA-MIBC **(A)**, GSE13507 **(B)**, GSE32894 **(C)**, GSE48075 **(D)**, CCLE-BLCA **(E)** datasets.

Supplementary Figure 12
**(A)** The mRNA expression of JAK/STAT3-related genes in SW780-control and RC48-exposed SW780 cells. **(B)** The plots showed the correlation between ERBB2 and STAT3 in mRNA levels in GSE13507, GSE48075, and GSE2894 datasets.

Supplementary Figure 13 Relationship between STAT3 and clinic. **(A)** Kaplain-Meier overall survival curve showed the prognosis difference of the MIBC patients in the high/low STAT3 mRNA expression group in the TCGA-MIBC dataset. **(B)** Kaplain-Meier recurrence-free survival curve showed survival differences of the MIBC patients in the high/low STAT3 expression group in the TCGA-MIBC dataset. **(C)** Kaplain-Meier overall survival curve showed survival differences of the MIBC patients in the high/low pSTAT3 (Tyr705) expression group in the TCGA-MIBC dataset. **(D)** Kaplain-Meier overall survival curve showed survival differences of the MIBC patients in the high/low STAT3 expression group in the GSE32894 dataset. **(E)** The box plot shows the STAT3 mRNA expression in different grades, gender, age, and stage.

Supplementary Figure 14 Artesunate could inhibit cell proliferation and reverse STAT3/pSTAT3 expression activated by RC48-exposed. **(A)** Colon formation assay reveals the cell growth after exposing different concentrations of ART. The percentage of survival cells was quantified. **(B)** Cell viability of luminal and basal cells after exposing ART (10 μM) at different times. **(C, D)** Western blot shows STAT3/pSTAT3 activated by the RC48-exposed could be reversed by ART.

Supplementary Figure 15 STAT3 inhibitors show a synergistic effect with RC48 against basal cells. **(A)** The colon formation assay of T24 and 5637 was exposed to saline, an individual drug (RC48 or BR-1-102), and a combination of the drugs. **(B)** The colon formation assay of T24 and 5637 was exposed to saline, an individual drug (RC48 or Stattic), and a combination of the drugs. **(C, E)** The CCK-8 proliferation assay of T24 and 5637 was exposed to saline, an individual drug (RC48 or BP-1-102), and a combination of the drugs. **(D, F)** The colon formation assay of T24 and 5637 was exposed to saline, an individual drug (RC48 or Stattic), and a combination of the drugs.
