# Supplementary material for: The combination treatment of RC48 and STAT3 inhibitor acts as a promising therapeutic strategy for basal bladder cancer
Source: Front Immunol. 2025 Jan 7;15:1432586. doi: 10.3389/fimmu.2024.1432586 (PMC11747467; doi:10.3389/fimmu.2024.1432586)
Supplement: Supplementary Figure 1 — Kaplan-Meier survival curve reveals the survival probability of the MIBC types based on different type classification systems, UNC subtype (A), MDA subtype (B), Consensus subtype (C), and TCGA subtype (D). [file DataSheet1.pdf]

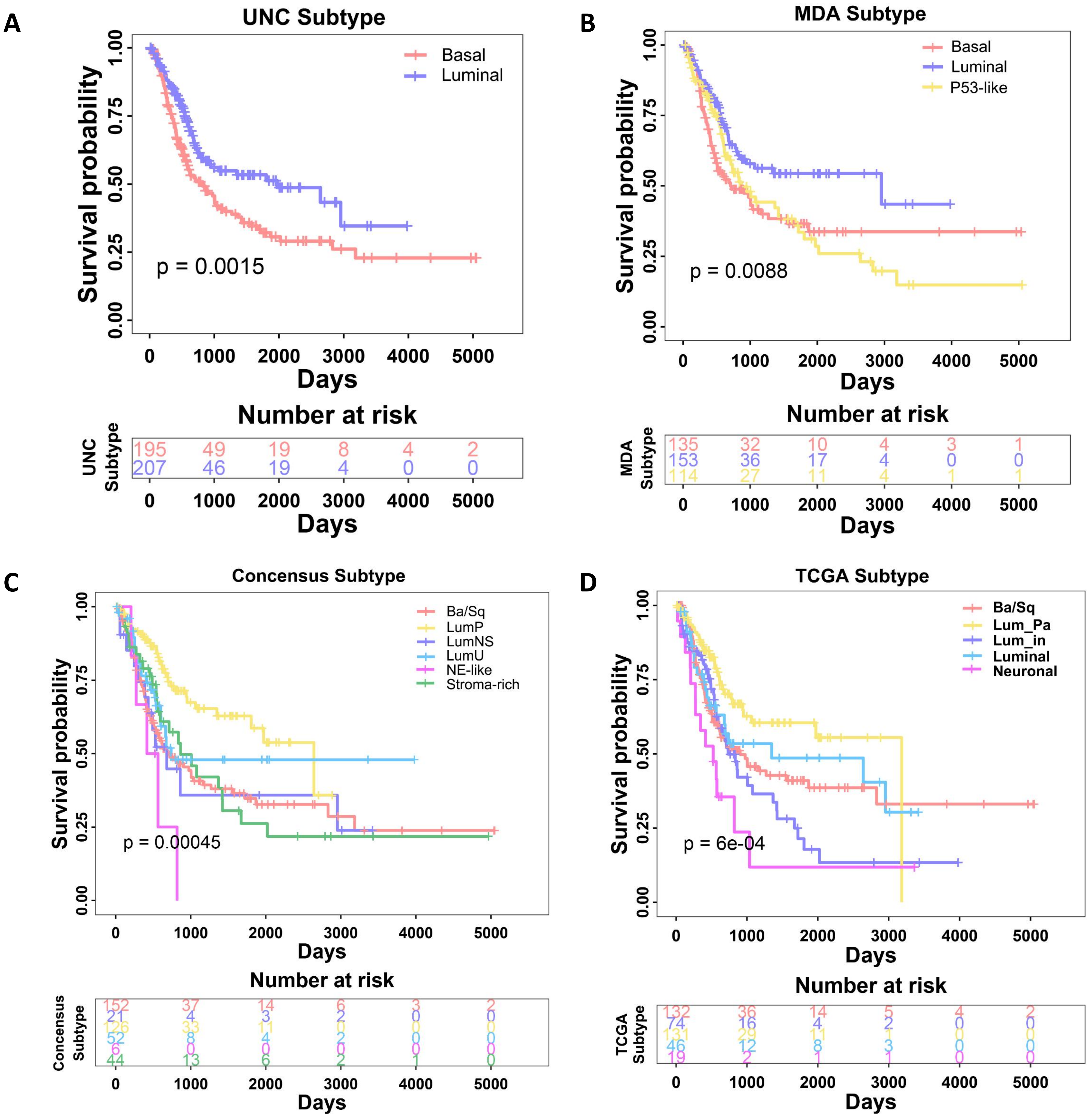
A
UNC Subtype
Basal
Luminal
p = 0.0015
Survival probability
Days
Number at risk
UNC Subtype
195 49 19 8 4 2
207 46 19 4 0 0
B
MDA Subtype
Basal
Luminal
P53-like
p = 0.0088
Survival probability
Days
Number at risk
MDA Subtype
135 32 10 4 3 1
153 36 17 4 0 0
114 27 11 4 1 1
C
Concensus Subtype
Ba/Sq
LumP
LumNS
LumU
NE-like
Stroma-rich
p = 0.00045
Survival probability
Days
Number at risk
Concensus Subtype
152 37 14 6 3 2
21 4 3 2 0 0
126 33 11 0 0 0
52 8 4 2 0 0
6 0 0 0 0 0
44 13 6 2 1 0
D
TCGA Subtype
Ba/Sq
Lum_Pa
Lum_in
Luminal
Neuronal
p = 6e-04
Survival probability
Days
Number at risk
TCGA Subtype
132 36 14 5 4 2
74 16 4 2 0 0
131 29 11 1 0 0
46 12 8 3 0 0
19 2 1 1 0 0

Figure S1

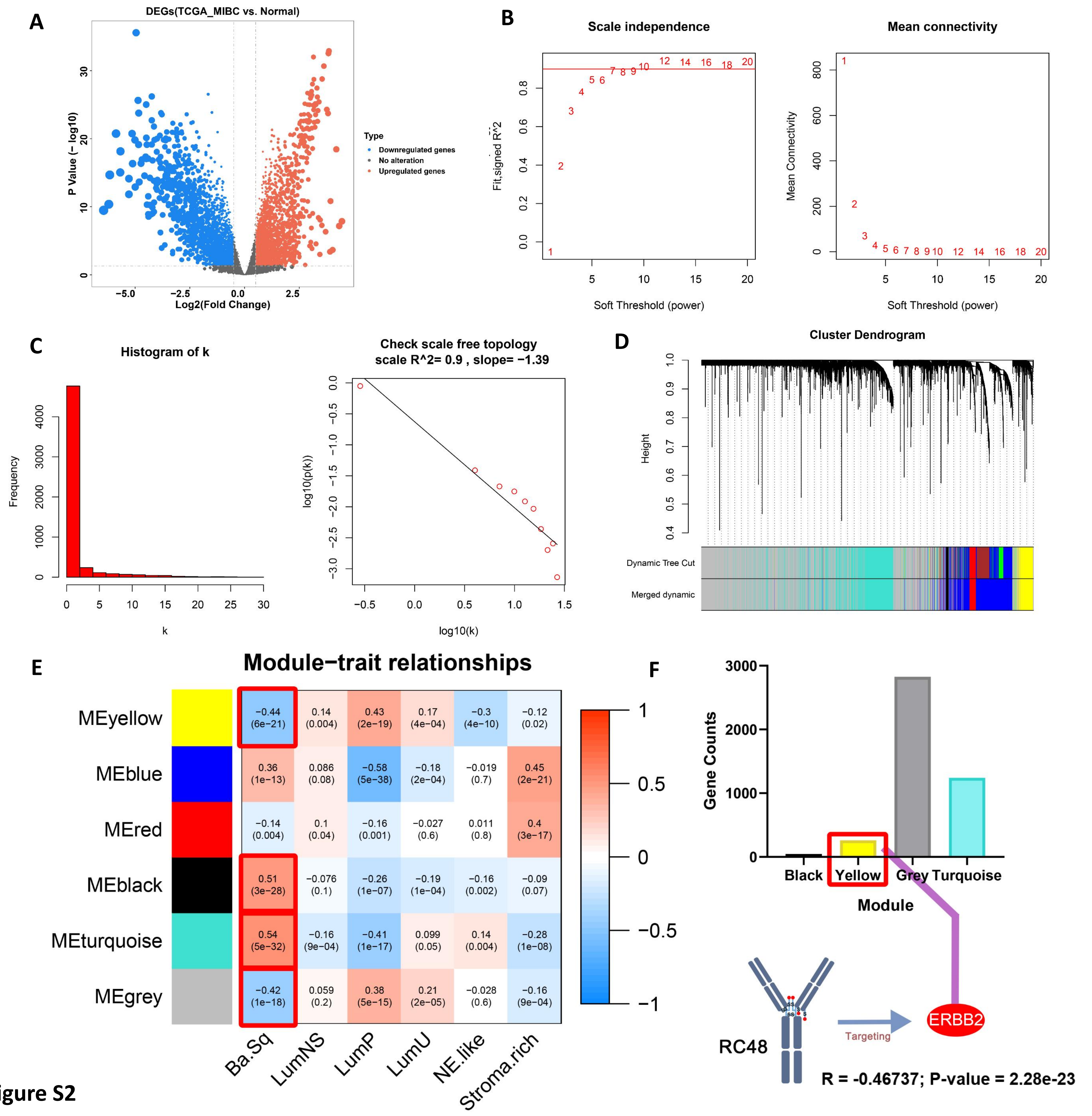
A
DEGs(TCGA_MIBC vs. Normal)
P Value (− log10)
Log2(Fold Change)
Type
Downregulated genes
No alteration
Upregulated genes
B
Scale independence
Fit,signed R^2
Soft Threshold (power)
Mean connectivity
Mean Connectivity
Soft Threshold (power)
C
Histogram of k
Frequency
k
Check scale free topology
scale R^2= 0.9 , slope= −1.39
log10(p(k))
log10(k)
D
Cluster Dendrogram
Height
Dynamic Tree Cut
Merged dynamic
E
Module−trait relationships
MEyellow
−0.44 (6e−21) | 0.14 (0.004) | 0.43 (2e−19) | 0.17 (4e−04) | −0.3 (4e−10) | −0.12 (0.02)
MEblue
0.36 (1e−13) | 0.086 (0.08) | −0.58 (5e−38) | −0.18 (2e−04) | −0.019 (0.7) | 0.45 (2e−21)
MEred
−0.14 (0.004) | 0.1 (0.04) | −0.16 (0.001) | −0.027 (0.6) | 0.011 (0.8) | 0.4 (3e−17)
MEblack
0.51 (3e−28) | −0.076 (0.1) | −0.26 (1e−07) | −0.19 (1e−04) | −0.16 (0.002) | −0.09 (0.07)
MEturquoise
0.54 (5e−32) | −0.16 (9e−04) | −0.41 (1e−17) | 0.099 (0.05) | 0.14 (0.004) | −0.28 (1e−08)
MEgrey
−0.42 (1e−18) | 0.059 (0.2) | 0.38 (5e−15) | 0.21 (2e−05) | −0.028 (0.6) | −0.16 (9e−04)
Ba.Sq
LumNS
LumP
LumU
NE.like
Stroma.rich
F
Gene Counts
Black
Yellow
Grey
Turquoise
Module
RC48
Targeting
ERBB2
R = -0.46737; P-value = 2.28e-23

Figure S2

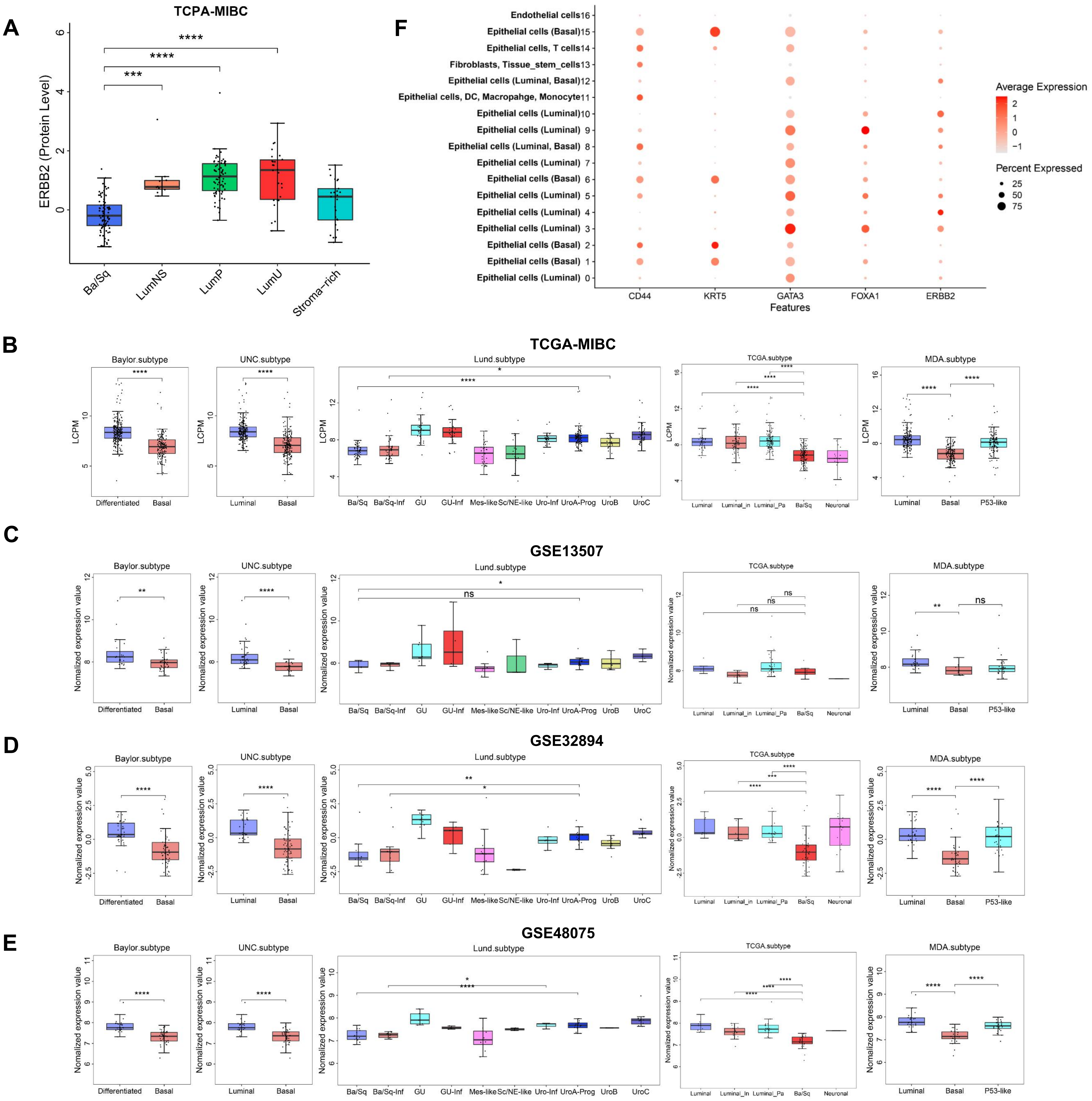
A
TCPA-MIBC
ERBB2 (Protein Level)
Ba/Sq
LumNS
LumP
LumU
Stroma-rich
F
Endothelial cells16
Epithelial cells (Basal)15
Epithelial cells, T cells14
Fibroblasts, Tissue_stem_cells13
Epithelial cells (Luminal, Basal)12
Epithelial cells, DC, Macropahge, Monocyte11
Epithelial cells (Luminal)10
Epithelial cells (Luminal) 9
Epithelial cells (Luminal, Basal) 8
Epithelial cells (Luminal) 7
Epithelial cells (Basal) 6
Epithelial cells (Luminal) 5
Epithelial cells (Luminal) 4
Epithelial cells (Luminal) 3
Epithelial cells (Basal) 2
Epithelial cells (Basal) 1
Epithelial cells (Luminal) 0
CD44
KRT5
GATA3
FOXA1
ERBB2
Features
Average Expression
Percent Expressed
B
TCGA-MIBC
Baylor.subtype
UNC.subtype
Lund.subtype
TCGA.subtype
MDA.subtype
LCPM
Differentiated
Basal
Luminal
Ba/Sq
Ba/Sq-Inf
GU
GU-Inf
Mes-like
Sc/NE-like
Uro-Inf
UroA-Prog
UroB
UroC
Luminal_in
Luminal_Pa
Neuronal
P53-like
C
GSE13507
Nomalized expression value
D
GSE32894
E
GSE48075

Figure S3

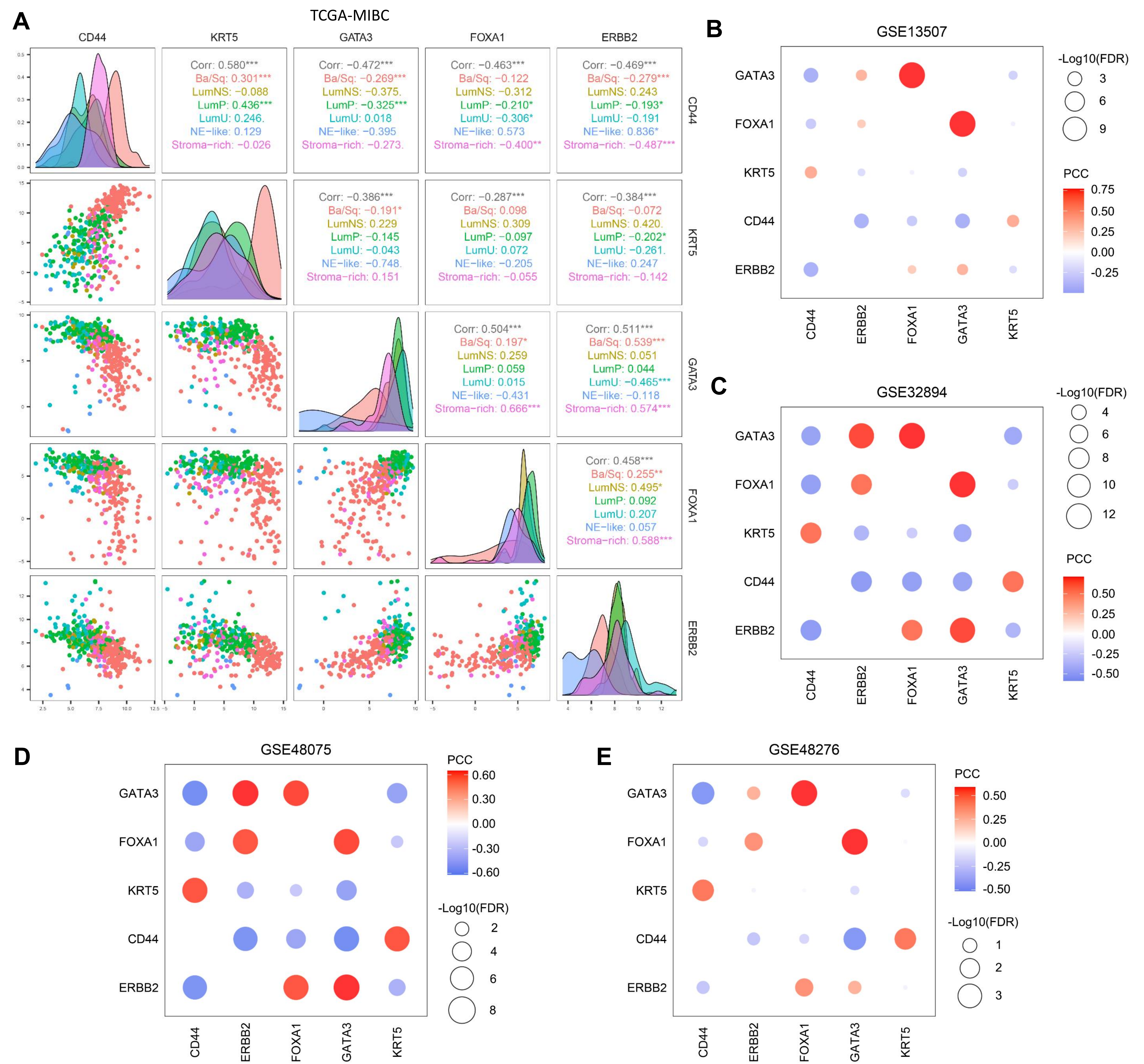
A
TCGA-MIBC
CD44
KRT5
GATA3
FOXA1
ERBB2
Corr: 0.580***
Ba/Sq: 0.301***
LumNS: −0.088
LumP: 0.436***
LumU: 0.246.
NE−like: 0.129
Stroma−rich: −0.026
Corr: −0.472***
Ba/Sq: −0.269***
LumNS: −0.375.
LumP: −0.325***
LumU: 0.018
NE−like: −0.395
Stroma−rich: −0.273.
Corr: −0.463***
Ba/Sq: −0.122
LumNS: −0.312
LumP: −0.210*
LumU: −0.306*
NE−like: 0.573
Stroma−rich: −0.400**
Corr: −0.469***
Ba/Sq: −0.279***
LumNS: 0.243
LumP: −0.193*
LumU: −0.191
NE−like: 0.836*
Stroma−rich: −0.487***
Corr: −0.386***
Ba/Sq: −0.191*
LumNS: 0.229
LumP: −0.145
LumU: −0.043
NE−like: −0.748.
Stroma−rich: 0.151
Corr: −0.287***
Ba/Sq: 0.098
LumNS: 0.309
LumP: −0.097
LumU: 0.072
NE−like: −0.205
Stroma−rich: −0.055
Corr: −0.384***
Ba/Sq: −0.072
LumNS: 0.420.
LumP: −0.202*
LumU: −0.261.
NE−like: 0.247
Stroma−rich: −0.142
Corr: 0.504***
Ba/Sq: 0.197*
LumNS: 0.259
LumP: 0.059
LumU: 0.015
NE−like: −0.431
Stroma−rich: 0.666***
Corr: 0.511***
Ba/Sq: 0.539***
LumNS: 0.051
LumP: 0.044
LumU: −0.465***
NE−like: −0.118
Stroma−rich: 0.574***
Corr: 0.458***
Ba/Sq: 0.255**
LumNS: 0.495*
LumP: 0.092
LumU: 0.207
NE−like: 0.057
Stroma−rich: 0.588***
B
GSE13507
-Log10(FDR)
3
6
9
PCC
0.75
0.50
0.25
0.00
-0.25
GATA3
FOXA1
KRT5
CD44
ERBB2
CD44
ERBB2
FOXA1
GATA3
KRT5
C
GSE32894
-Log10(FDR)
4
6
8
10
12
PCC
0.50
0.25
0.00
-0.25
-0.50
D
GSE48075
PCC
0.60
0.30
0.00
-0.30
-0.60
-Log10(FDR)
2
4
6
8
E
GSE48276
PCC
0.50
0.25
0.00
-0.25
-0.50
-Log10(FDR)
1
2
3

Figure S4

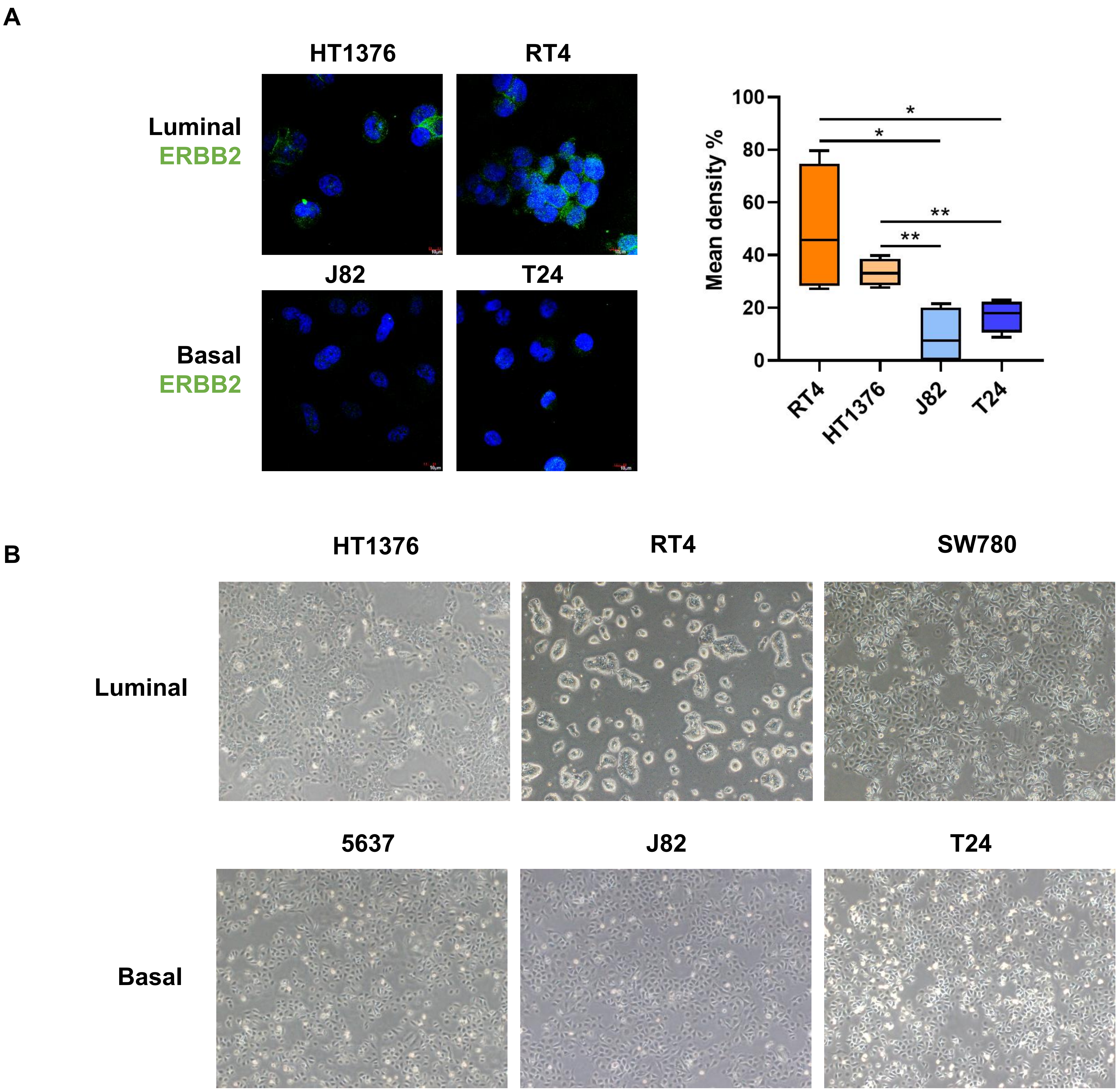
A
HT1376
RT4
Luminal
ERBB2
J82
T24
Basal
ERBB2
Mean density %
100
80
60
40
20
0
*
*
**
**
RT4
HT1376
J82
T24
B
HT1376
RT4
SW780
Luminal
5637
J82
T24
Basal

Figure S5

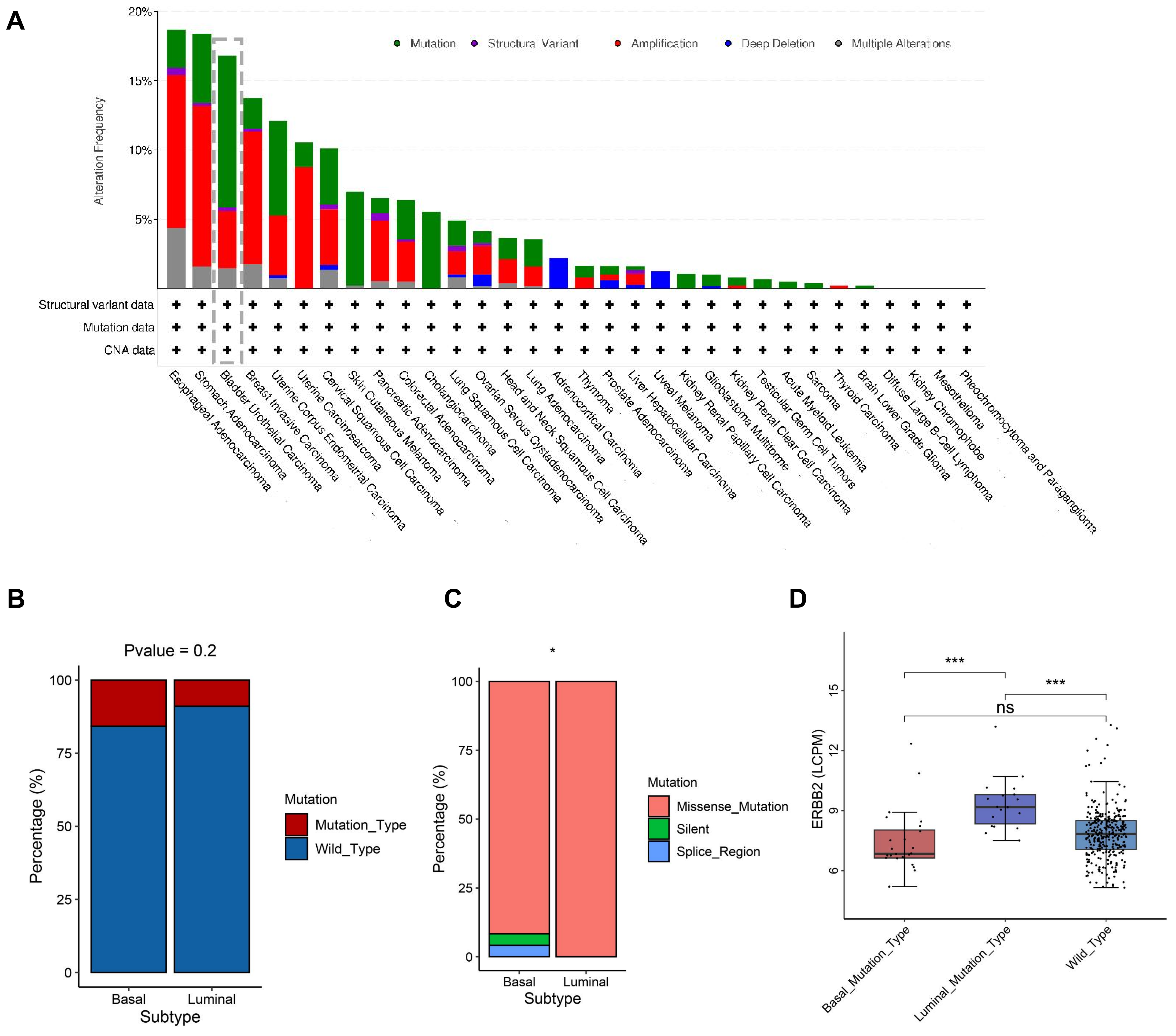
A
20%
15%
10%
5%
Alteration Frequency
Mutation
Structural Variant
Amplification
Deep Deletion
Multiple Alterations
Structural variant data
Mutation data
CNA data
Esophageal Adenocarcinoma
Stomach Adenocarcinoma
Bladder Urothelial Carcinoma
Breast Invasive Carcinoma
Uterine Carcinosarcoma
Uterine Corpus Endometrial Carcinoma
Cervical Squamous Cell Carcinoma
Skin Cutaneous Melanoma
Pancreatic Adenocarcinoma
Colorectal Adenocarcinoma
Cholangiocarcinoma
Lung Squamous Cell Carcinoma
Ovarian Serous Cystadenocarcinoma
Head and Neck Squamous Cell Carcinoma
Lung Adenocarcinoma
Adrenocortical Carcinoma
Thymoma
Prostate Adenocarcinoma
Liver Hepatocellular Carcinoma
Uveal Melanoma
Kidney Renal Papillary Cell Carcinoma
Glioblastoma Multiforme
Kidney Renal Clear Cell Carcinoma
Testicular Germ Cell Tumors
Acute Myeloid Leukemia
Sarcoma
Thyroid Carcinoma
Brain Lower Grade Glioma
Diffuse Large B-Cell Lymphoma
Kidney Chromophobe
Mesothelioma
Pheochromocytoma and Paraganglioma
B
Pvalue = 0.2
Percentage (%)
100
75
50
25
0
Mutation
Mutation_Type
Wild_Type
Basal
Luminal
Subtype
C
*
Percentage (%)
100
75
50
25
0
Mutation
Missense_Mutation
Silent
Splice_Region
Basal
Luminal
Subtype
D
***
***
ns
ERBB2 (LCPM)
15
12
9
6
Basal_Mutation_Type
Luminal_Mutation_Type
Wild_Type

Figure S6

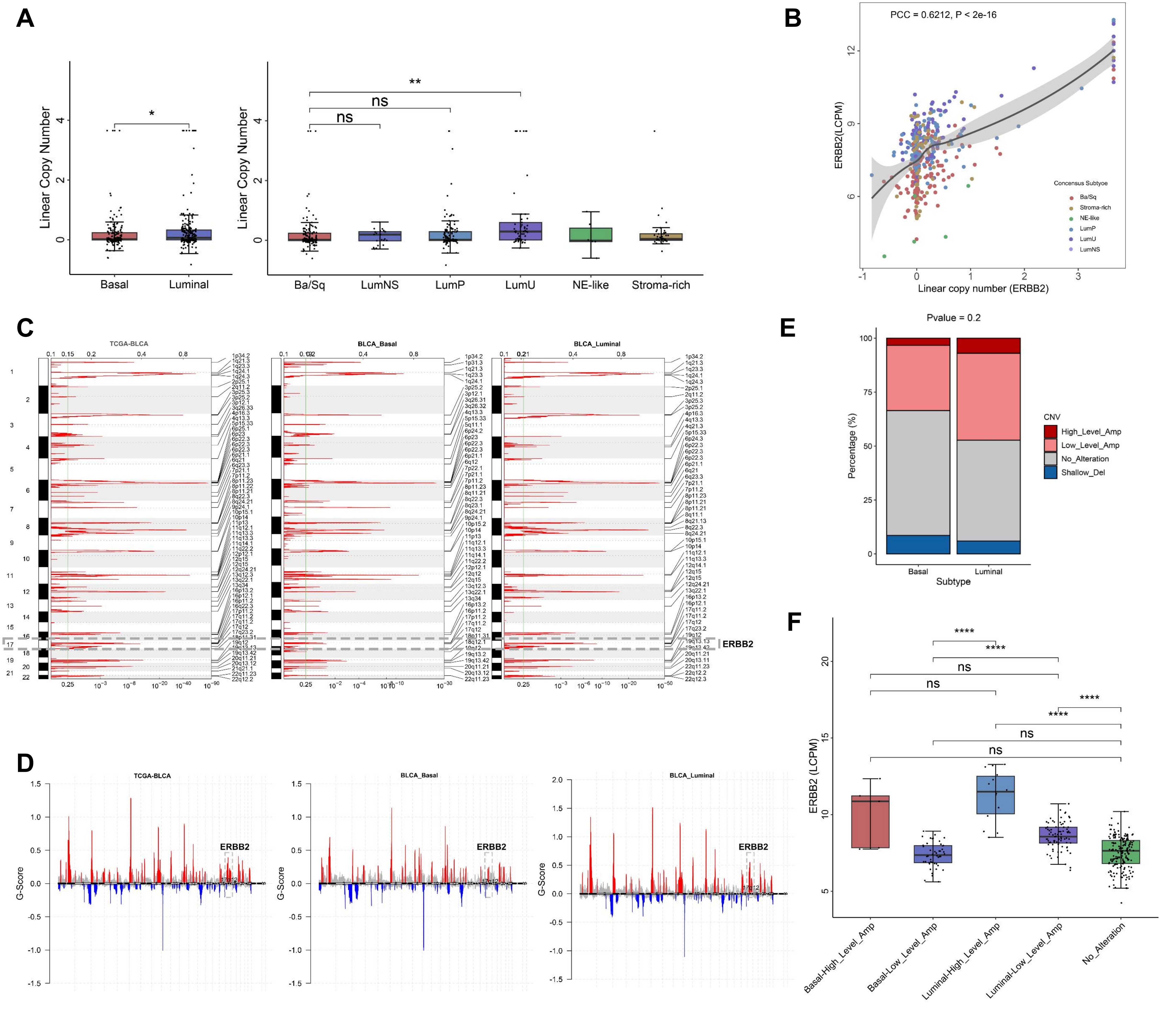
A
Linear Copy Number
Basal
Luminal
Ba/Sq
LumNS
LumP
LumU
NE-like
Stroma-rich
B
PCC = 0.6212, P < 2e-16
ERBB2(LCPM)
Linear copy number (ERBB2)
Concensus Subtyoe
Ba/Sq
Stroma-rich
NE-like
LumP
LumU
LumNS
C
TCGA-BLCA
BLCA_Basal
BLCA_Luminal
ERBB2
D
TCGA-BLCA
BLCA_Basal
BLCA_Luminal
G-Score
ERBB2
E
Pvalue = 0.2
Percentage (%)
CNV
High_Level_Amp
Low_Level_Amp
No_Alteration
Shallow_Del
Basal
Luminal
Subtype
F
ERBB2 (LCPM)
Basal-High_Level_Amp
Basal-Low_Level_Amp
Luminal-High_Level_Amp
Luminal-Low_Level_Amp
No_Alteration

Figure S7

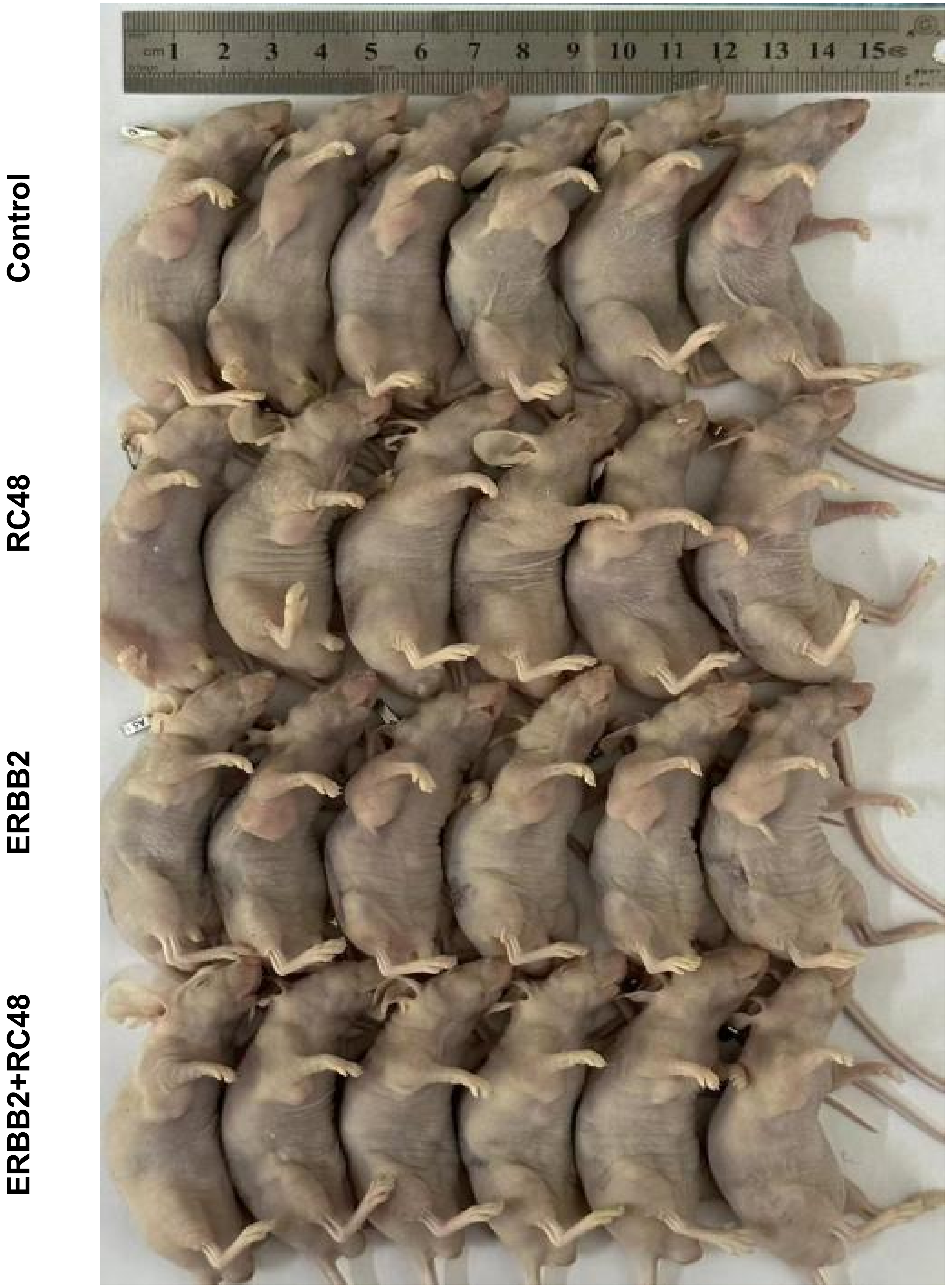

Figure S8

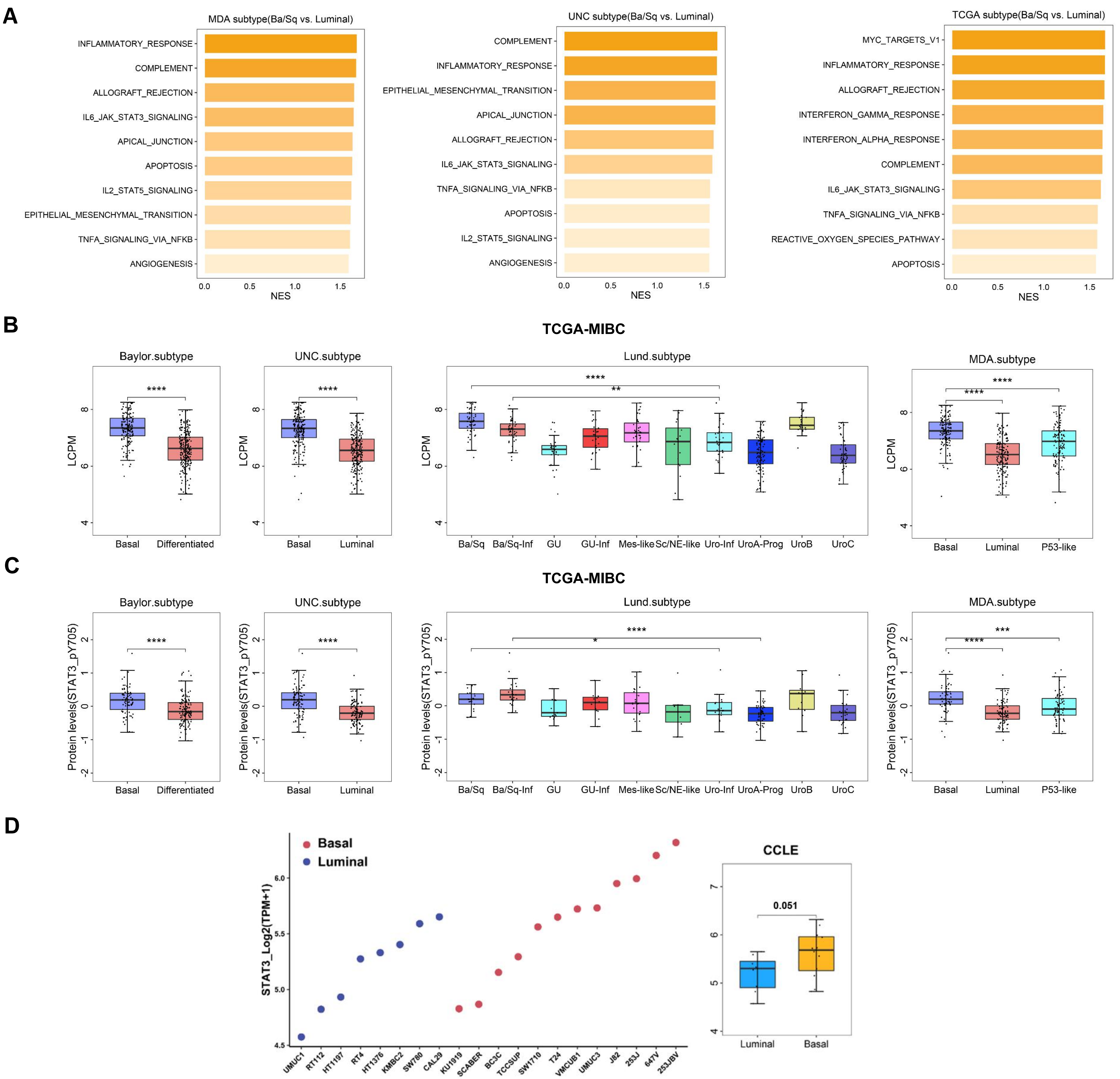
A
MDA subtype(Ba/Sq vs. Luminal)
INFLAMMATORY_RESPONSE
COMPLEMENT
ALLOGRAFT_REJECTION
IL6_JAK_STAT3_SIGNALING
APICAL_JUNCTION
APOPTOSIS
IL2_STAT5_SIGNALING
EPITHELIAL_MESENCHYMAL_TRANSITION
TNFA_SIGNALING_VIA_NFKB
ANGIOGENESIS
NES
UNC subtype(Ba/Sq vs. Luminal)
COMPLEMENT
INFLAMMATORY_RESPONSE
EPITHELIAL_MESENCHYMAL_TRANSITION
APICAL_JUNCTION
ALLOGRAFT_REJECTION
IL6_JAK_STAT3_SIGNALING
TNFA_SIGNALING_VIA_NFKB
APOPTOSIS
IL2_STAT5_SIGNALING
ANGIOGENESIS
NES
TCGA subtype(Ba/Sq vs. Luminal)
MYC_TARGETS_V1
INFLAMMATORY_RESPONSE
ALLOGRAFT_REJECTION
INTERFERON_GAMMA_RESPONSE
INTERFERON_ALPHA_RESPONSE
COMPLEMENT
IL6_JAK_STAT3_SIGNALING
TNFA_SIGNALING_VIA_NFKB
REACTIVE_OXYGEN_SPECIES_PATHWAY
APOPTOSIS
NES
B
TCGA-MIBC
Baylor.subtype
UNC.subtype
Lund.subtype
MDA.subtype
LCPM
Basal
Differentiated
Luminal
Ba/Sq
Ba/Sq-Inf
GU
GU-Inf
Mes-like
Sc/NE-like
Uro-Inf
UroA-Prog
UroB
UroC
P53-like
C
TCGA-MIBC
Protein levels(STAT3_pY705)
D
Basal
Luminal
STAT3_Log2(TPM+1)
UMUC1
RT112
HT1197
RT4
HT1376
KMBC2
SW780
CAL29
KU1919
SCABER
BC3C
TCCSUP
SW1710
T24
VMCUB1
UMUC3
J82
253J
647V
253JBV
CCLE
0.051

Figure S9

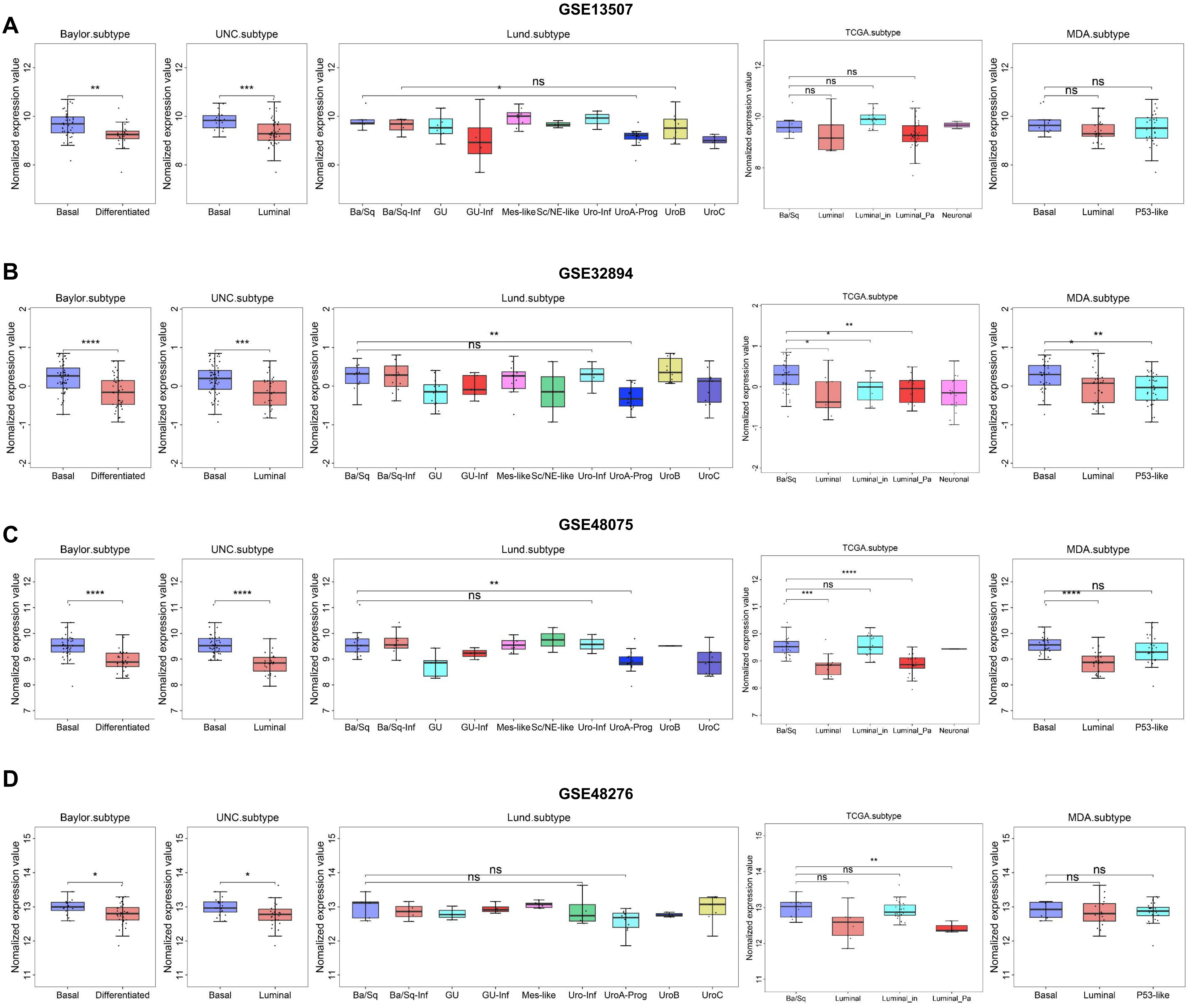

A
GSE13507
Baylor.subtype
UNC.subtype
Lund.subtype
TCGA.subtype
MDA.subtype
Nomalized expression value
Basal
Differentiated
Luminal
Ba/Sq
Ba/Sq-Inf
GU
GU-Inf
Mes-like
Sc/NE-like
Uro-Inf
UroA-Prog
UroB
UroC
Luminal_in
Luminal_Pa
Neuronal
P53-like
B
GSE32894
C
GSE48075
D
GSE48276
ns

Figure S10

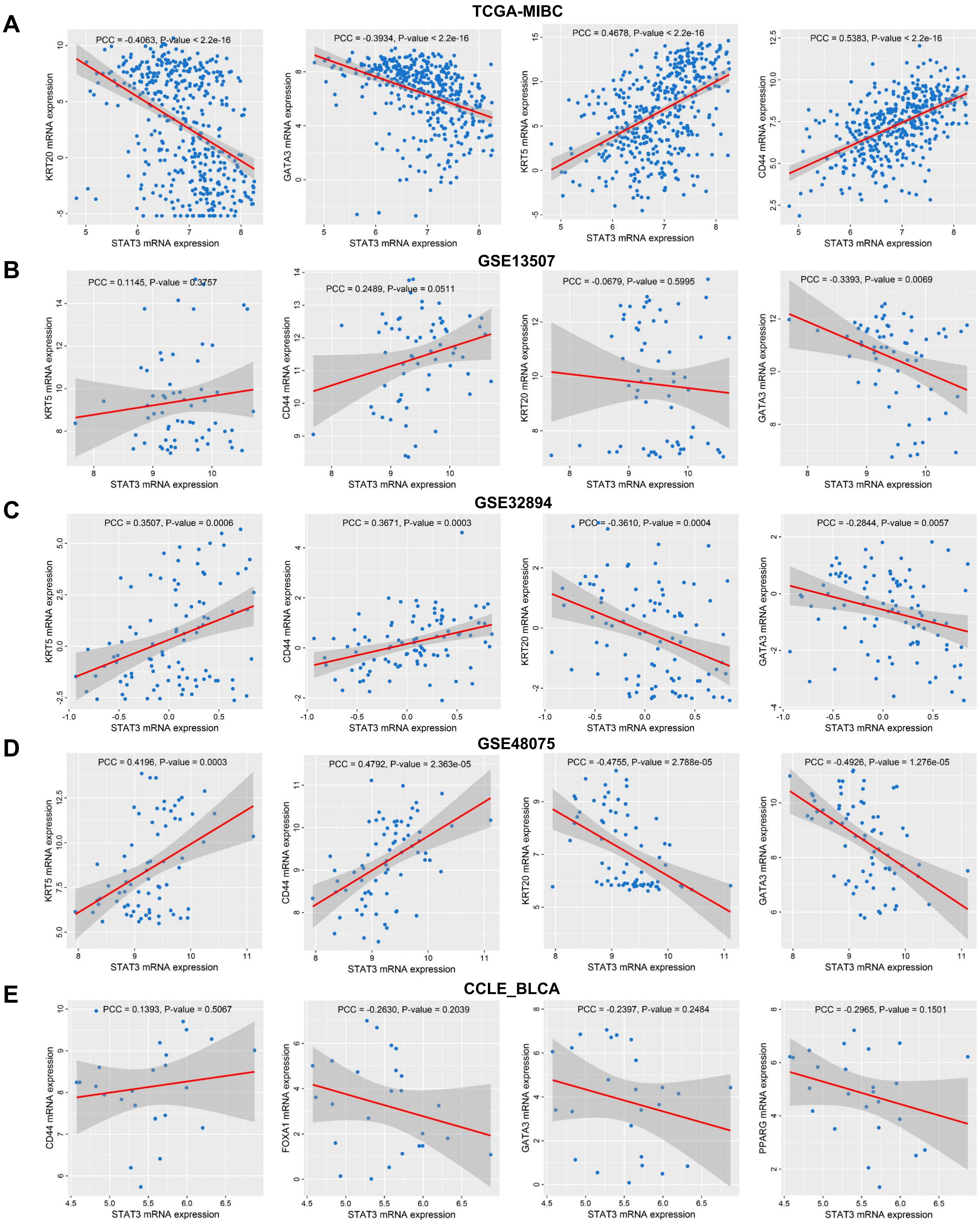
TCGA-MIBC
A
PCC = -0.4063, P-value < 2.2e-16
KRT20 mRNA expression
STAT3 mRNA expression
PCC = -0.3934, P-value < 2.2e-16
GATA3 mRNA expression
STAT3 mRNA expression
PCC = 0.4678, P-value < 2.2e-16
KRT5 mRNA expression
STAT3 mRNA expression
PCC = 0.5383, P-value < 2.2e-16
CD44 mRNA expression
STAT3 mRNA expression
GSE13507
B
PCC = 0.1145, P-value = 0.3757
KRT5 mRNA expression
STAT3 mRNA expression
PCC = 0.2489, P-value = 0.0511
CD44 mRNA expression
STAT3 mRNA expression
PCC = -0.0679, P-value = 0.5995
KRT20 mRNA expression
STAT3 mRNA expression
PCC = -0.3393, P-value = 0.0069
GATA3 mRNA expression
STAT3 mRNA expression
GSE32894
C
PCC = 0.3507, P-value = 0.0006
KRT5 mRNA expression
STAT3 mRNA expression
PCC = 0.3671, P-value = 0.0003
CD44 mRNA expression
STAT3 mRNA expression
PCC = -0.3610, P-value = 0.0004
KRT20 mRNA expression
STAT3 mRNA expression
PCC = -0.2844, P-value = 0.0057
GATA3 mRNA expression
STAT3 mRNA expression
GSE48075
D
PCC = 0.4196, P-value = 0.0003
KRT5 mRNA expression
STAT3 mRNA expression
PCC = 0.4792, P-value = 2.363e-05
CD44 mRNA expression
STAT3 mRNA expression
PCC = -0.4755, P-value = 2.788e-05
KRT20 mRNA expression
STAT3 mRNA expression
PCC = -0.4926, P-value = 1.276e-05
GATA3 mRNA expression
STAT3 mRNA expression
CCLE_BLCA
E
PCC = 0.1393, P-value = 0.5067
CD44 mRNA expression
STAT3 mRNA expression
PCC = -0.2630, P-value = 0.2039
FOXA1 mRNA expression
STAT3 mRNA expression
PCC = -0.2397, P-value = 0.2484
GATA3 mRNA expression
STAT3 mRNA expression
PCC = -0.2965, P-value = 0.1501
PPARG mRNA expression
STAT3 mRNA expression

Figure S11

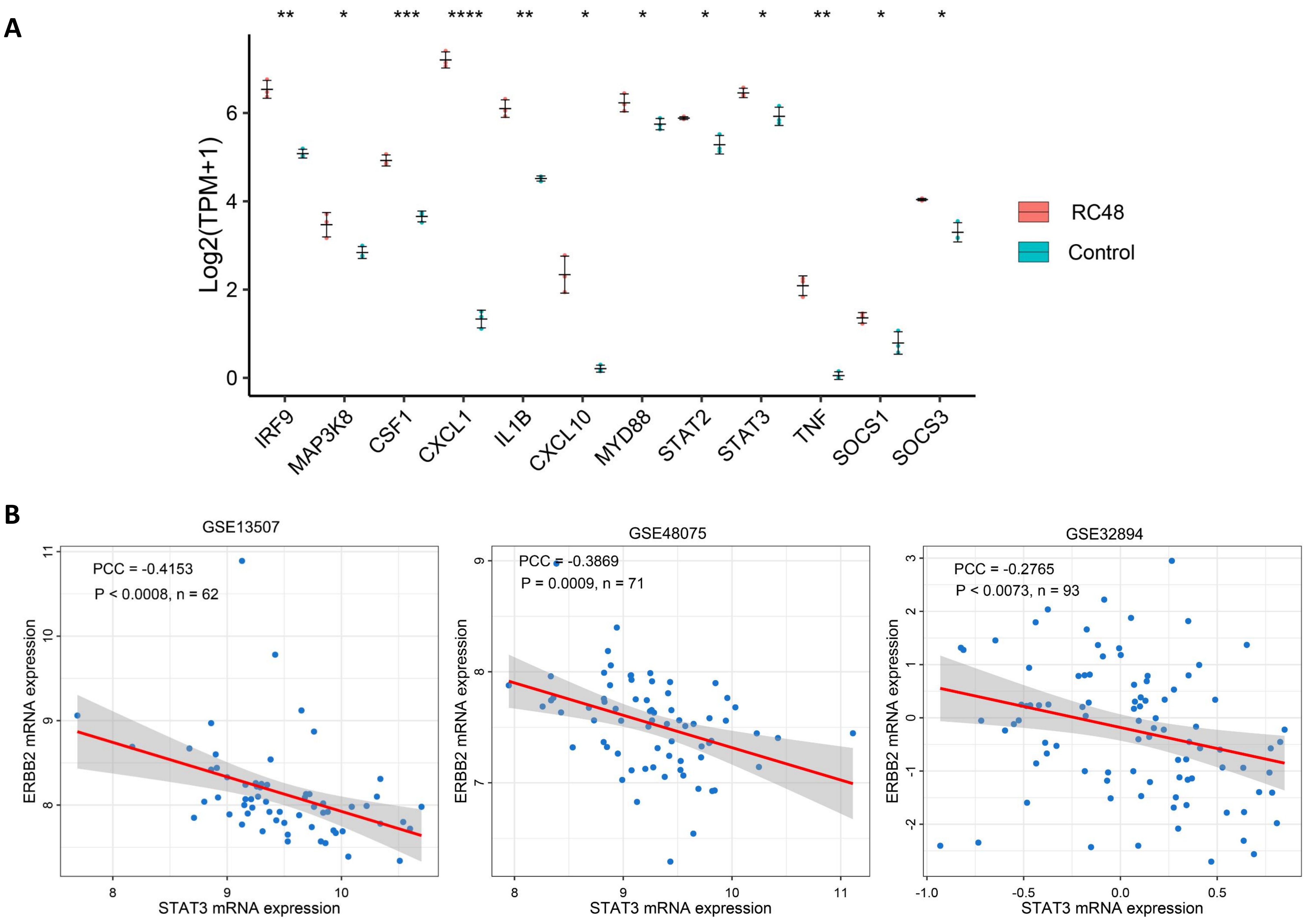
A
** * *** **** ** * * * * ** * *
Log2(TPM+1)
0
2
4
6
RC48
Control
IRF9
MAP3K8
CSF1
CXCL1
IL1B
CXCL10
MYD88
STAT2
STAT3
TNF
SOCS1
SOCS3
B
GSE13507
PCC = -0.4153
P < 0.0008, n = 62
ERBB2 mRNA expression
STAT3 mRNA expression
GSE48075
PCC = -0.3869
P = 0.0009, n = 71
ERBB2 mRNA expression
STAT3 mRNA expression
GSE32894
PCC = -0.2765
P < 0.0073, n = 93
ERBB2 mRNA expression
STAT3 mRNA expression

Figure S12

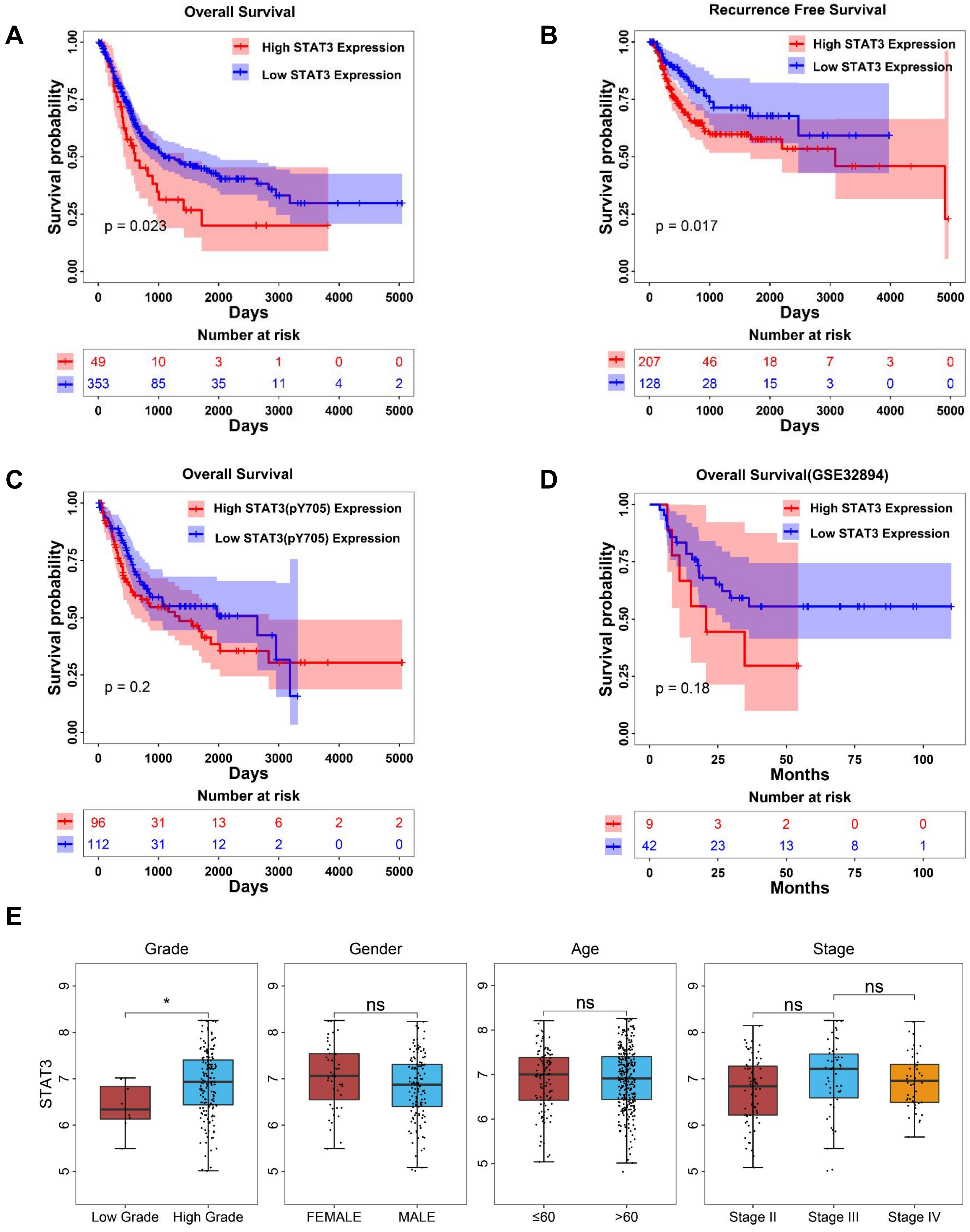
A
Overall Survival
High STAT3 Expression
Low STAT3 Expression
Survival probability
p = 0.023
Days
Number at risk
49 10 3 1 0 0
353 85 35 11 4 2
B
Recurrence Free Survival
High STAT3 Expression
Low STAT3 Expression
p = 0.017
207 46 18 7 3 0
128 28 15 3 0 0
C
Overall Survival
High STAT3(pY705) Expression
Low STAT3(pY705) Expression
p = 0.2
96 31 13 6 2 2
112 31 12 2 0 0
D
Overall Survival(GSE32894)
High STAT3 Expression
Low STAT3 Expression
p = 0.18
Months
9 3 2 0 0
42 23 13 8 1
E
Grade
Gender
Age
Stage
STAT3
*
ns
Low Grade
High Grade
FEMALE
MALE
≤60
>60
Stage II
Stage III
Stage IV

Figure S13

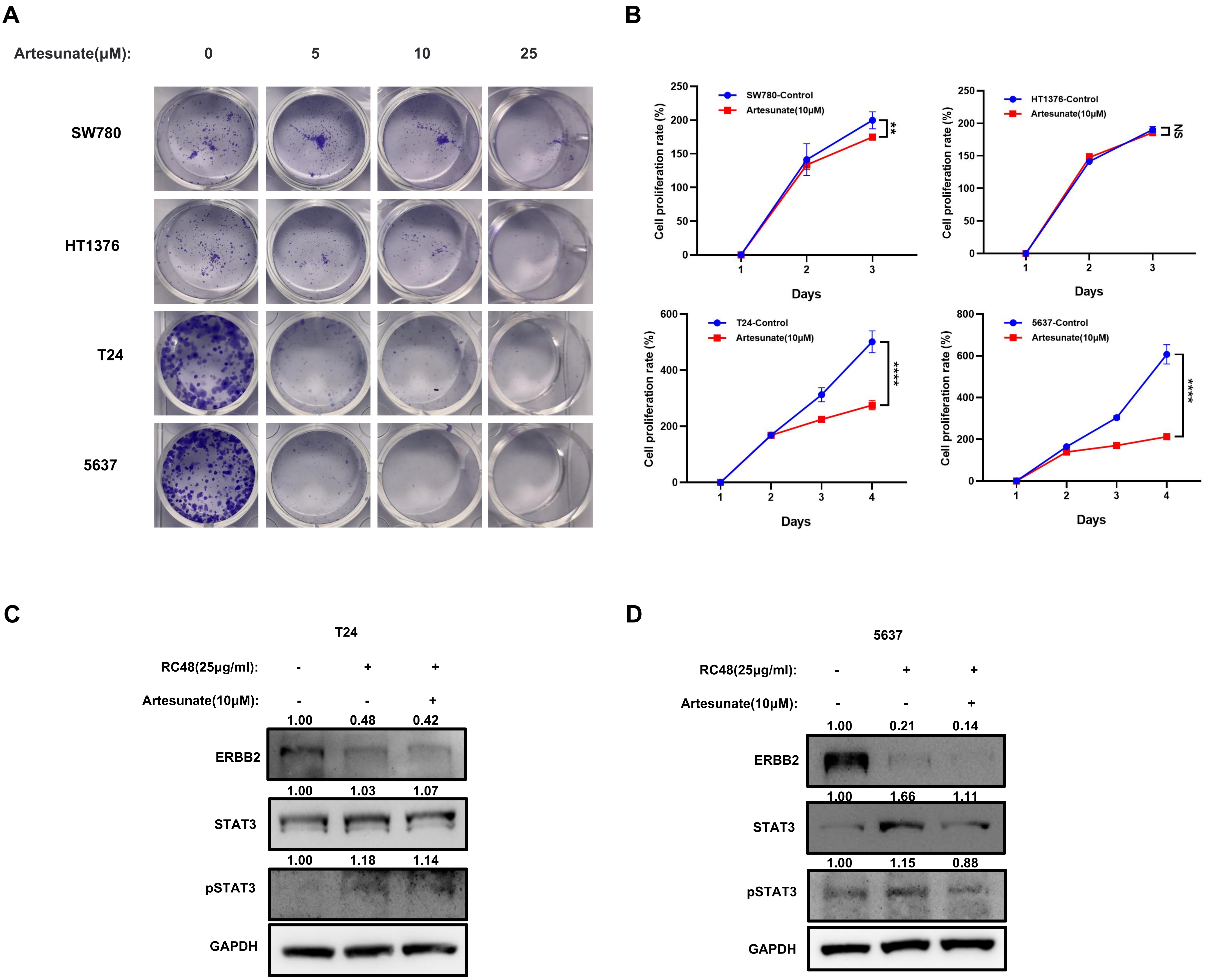
A
Artesunate(μM):
0
5
10
25
SW780
HT1376
T24
5637
B
Cell proliferation rate (%)
SW780-Control
Artesunate(10μM)
**
Days
HT1376-Control
Artesunate(10μM)
NS
T24-Control
Artesunate(10μM)
****
5637-Control
Artesunate(10μM)
****
C
T24
RC48(25μg/ml): - + +
Artesunate(10μM): - - +
1.00 0.48 0.42
ERBB2
1.00 1.03 1.07
STAT3
1.00 1.18 1.14
pSTAT3
GAPDH
D
5637
RC48(25μg/ml): - + +
Artesunate(10μM): - - +
1.00 0.21 0.14
ERBB2
1.00 1.66 1.11
STAT3
1.00 1.15 0.88
pSTAT3
GAPDH

Figure S14

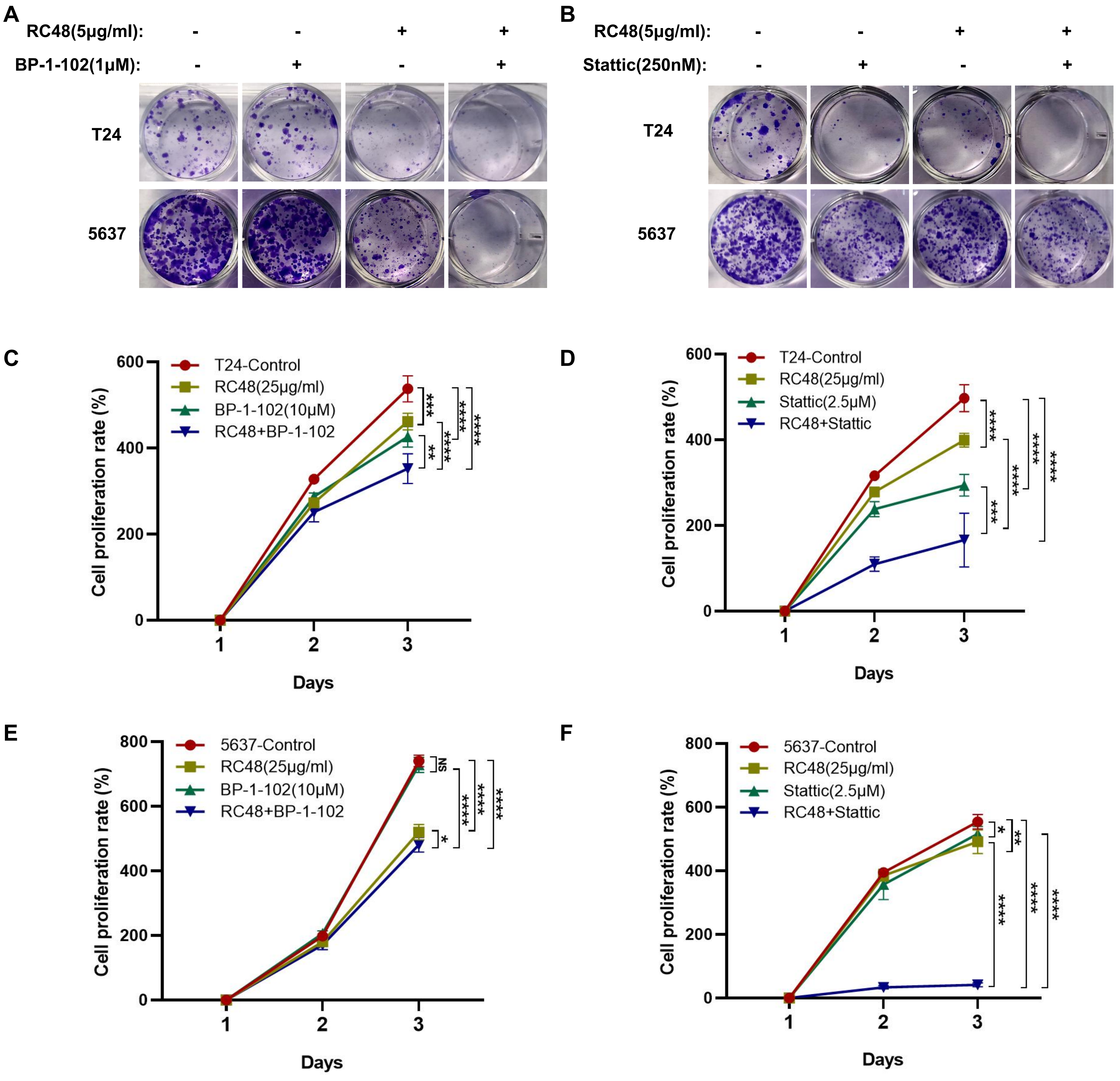
A
RC48(5μg/ml):
BP-1-102(1μM):
T24
5637
B
RC48(5μg/ml):
Stattic(250nM):
T24
5637
C
Cell proliferation rate (%)
Days
T24-Control
RC48(25μg/ml)
BP-1-102(10μM)
RC48+BP-1-102
D
Cell proliferation rate (%)
Days
T24-Control
RC48(25μg/ml)
Stattic(2.5μM)
RC48+Stattic
E
Cell proliferation rate (%)
Days
5637-Control
RC48(25μg/ml)
BP-1-102(10μM)
RC48+BP-1-102
F
Cell proliferation rate (%)
Days
5637-Control
RC48(25μg/ml)
Stattic(2.5μM)
RC48+Stattic

Figure S15
